# Effects of Neuraxial Blockade May Be Difficult To Study Using Large Randomized Controlled Trials: The PeriOperative Epidural Trial (POET) Pilot Study

**DOI:** 10.1371/journal.pone.0004644

**Published:** 2009-02-27

**Authors:** Peter T. Choi, W. Scott Beattie, Gregory L. Bryson, James E. Paul, Homer Yang

**Affiliations:** 1 Department of Anesthesiology, Pharmacology and Therapeutics, University of British Columbia, Vancouver, British Columbia, Canada; 2 Department of Anaesthesia, University Health Network, Toronto, Ontario, Canada; 3 Department of Anesthesiology, University of Ottawa, Ottawa, Ontario, Canada; 4 Department of Anesthesia, McMaster University, Hamilton, Ontario, Canada; Women's and Children's Hospital, Australia

## Abstract

**Background:**

Early randomized controlled trials have suggested that neuraxial blockade may reduce cardiorespiratory complications after non-cardiothoracic surgery, but recent larger trials have been inconclusive. We conducted a pilot study to assess the feasibility of conducting a large multicentre randomized controlled trial in Canada.

**Methodology/Principal Findings:**

After Research Ethics Board approvals from the participating institutions, subjects were recruited if they were ≥45 years old, had an expected hospital stay ≥48 hours, were undergoing a noncardiothoracic procedure amenable to epidural analgesia, met one of six risk criteria, and did not have contraindications to neuraxial blockade. After informed consent, subjects were randomly allocated to combined epidural analgesia (epidural group) and neuraxial anesthesia, with or without general anesthesia, or intravenous opioid analgesia (IV group) and general anesthesia. The primary outcomes were the rate of recruitment and the percents of eligible patients recruited, crossed over, and followed completely. Feasibility targets were defined *a priori*. A blinded, independent committee adjudicated the secondary clinical outcomes. Subjects were followed daily while in hospital and then at 30 days after surgery. Analysis was intention-to-treat. Over a 15-month period, the recruitment rate was 0.5±0.3 (mean±SEM) subjects per week per centre; 112/494 (22.7%) eligible subjects were recruited at four tertiary-care teaching hospitals in Canada. Thirteen (26.5%) of 49 subjects in the epidural group crossed over to the IV group; seven (14.3%) were due to failed or inadequate analgesia or complications from epidural analgesia. Five (9.8%) of 51 subjects in the IV group crossed over to the epidural group but none were due to inadequate analgesia or complications. Ninety-eight (97.0%) of 101 subjects were successfully followed up until 30 days after their surgery.

**Conclusion/Significance:**

Of the criteria we defined for the feasibility of a full-scale trial, only the follow-up target was met. The other feasibility outcomes did not meet our preset criteria for success. The results suggest that a large multicentre trial may not be a feasible design to study the perioperative effects of neuraxial blockade.

**Trial Registration:**

ClinicalTrials.gov NCT 0221260

Controlled-Trials.com ISRCTN 35629817

## Introduction

Perioperative cardiorespiratory events are frequent complications of non-cardiothoracic surgery and result in significant morbidity, mortality, and cost [1]–[3]. Mortality after noncardiac surgery ranges from 0.3% to 3.6% depending on the level of risk [4], [5]. The incidence has remained unchanged over the past decade [4]. In one national study of perioperative outcomes and death, cardiac failure, respiratory complications, and cardiac arrest were the most frequent postoperative complications [6]. Moderate-sized prospective cohort studies report that in patients with or at risk of cardiac disease undergoing non-cardiac surgery, 2.4% to 5.8% will suffer a major cardiac event [2]. Rates of postoperative pneumonia in non-cardiothoracic surgery vary from 9% to 40% [3], demonstrating both the heterogeneity in patients undergoing non-cardiothoracic surgery and the magnitude of the problem.

Results from randomized controlled trials (RCTs) and meta-analyses have suggested a number of potentially beneficial drugs (*e.g.*, beta-blockers, calcium channel blockers, statins, alpha-2 agonists) to reduce perioperative cardiovascular events [7]–[11] and potentially beneficial interventions (*e.g.*, lung expansion maneuvers, selective nasogastric decompression) to reduce perioperative pulmonary events [12]. However, with the exception of the European MIvazerol Trial (EMIT) [13] and the PeriOperative ISchemia Evaluation (POISE) Study [14], the evidence is based on data from RCTs with sample sizes of less than 1000 subjects.

Considering the high frequency of cardiorespiratory complications following non-cardiothoracic surgery, an ideal intervention would reduce both cardiac and respiratory complications in a wide range of perioperative settings. Perioperative neuraxial blockade may well meet this criterion. Neuraxial blockade has potentially beneficial effects on the cardiovascular, hematological, and respiratory systems [15]. The resultant changes in catecholamines, prothrombotic mediators, and respiratory function begin during surgery and continue postoperatively; thus, maximally effective neuraxial blockade should be administered intraoperatively and continued postoperatively. The benefits achieved by neuraxial blockade should, in theory, reduce perioperative thrombotic (*e.g.*, myocardial infarction, deep vein thrombosis) and respiratory (*e.g.*, pneumonia, respiratory failure) events.

Several meta-analyses have evaluated the effect of neuraxial blockade on perioperative outcomes [16]–[20]. Based primarily on data from small studies, the meta-analyses found reductions in 30-day all-cause postoperative mortality (odds ratio [OR] 0.70; 95% confidence interval [CI] 0.54–0.90), deep vein thrombosis (OR 0.56; 95% CI 0.43–0.72), pulmonary embolism (OR 0.45; 95% CI 0.29–0.69), pneumonia (OR 0.61; 95% CI 0.48–0.76), and respiratory depression (OR 0.41; 95% 0.23–0.73) and showed a trend toward reduced postoperative myocardial infarction with the use of neuraxial blockade (OR 0.67; 95% CI 0.45–1.00) [16]. There was no difference in all-cause mortality or myocardial infarction between postoperative epidural analgesia and intravenous analgesia of at least 24 hour duration (OR 0.74; 0.40–1.37) [17]; however, there was a statistically significant reduction for myocardial infarction in patients receiving thoracic epidural analgesia (OR 0.64; 95% CI 0.42–0.97) [18]. Postoperative epidural analgesia with local anesthetics also reduced respiratory infections compared to systemic narcotics (relative risk 0.36; 95% CI 0.21–0.65) [19]. In summary, meta-analyses show consistent strong trends toward reduction in all-cause mortality with neuraxial anesthesia and analgesia. Data are consistent with decreases in cardiovascular and pulmonary events contributing to reductions in all-cause mortality, with both intraoperative and postoperative blockade playing a role in reducing mortality.

Subsequently, two moderate-size RCTs have examined the effect of perioperative neuraxial blockade in moderate- to high-risk patients undergoing major abdominal operations [21], [22]. Both RCTs compared combined epidural and general anesthesia and postoperative epidural analgesia to general anesthesia and postoperative intravenous (i.v.) opioid analgesia. In contrast to the meta-analyses, the two studies observed trends toward increased 30-day all-cause mortality in the epidural blockade groups (epidural 23/447, i.v. 19/441; *p* = 0.67) [21], (epidural 20/514, i.v. 17/507; *p* = 0.74) [22]. There were no differences in major postoperative morbidity in the neuraxial group with the exception of reduced respiratory failure overall (epidural 104/447, i.v. 133/441; *p* = 0.02) [21] and reduced nonfatal myocardial infarction in a subgroup of patients undergoing abdominal aortic surgery (epidural 5/184, i.v. 15/190; *p* = 0.05) [22].

The trends toward reduction in cardiorespiratory events with neuraxial blockade observed in meta-analyses and RCTs are encouraging, but the discrepancy between meta-analyses and moderate-size RCTs with regards to all-cause mortality raises doubts about the use of neuraxial blockade. Because of the susceptibility to publication bias, and previous instances in which subsequent trials contradicted results of meta-analyses of small trials, clinicians are legitimately skeptical about considering a meta-analysis of many small trials as providing definitive evidence. The numbers of subjects in clinical trials are still insufficient to detect clinically relevant differences in most perioperative complications [20]. A large RCT is needed to inform clinicians of the benefits and harm of neuraxial blockade with regards to perioperative cardiorespiratory events.

Prior to embarking on a large, multicentre trial we needed a pilot study to inform us of several key issues. The objectives of the PostOperative Epidural Trial (POET) Pilot Study were 1) to determine the feasibility of recruiting and following patients in a timely manner and 2) to estimate the incidence rate of the primary clinical outcome to be used in size calculations for a large definitive trial. Specifically, we wished to determine the recruitment rate across centres, the percent of eligible patients recruited, the rates of crossover from one analgesic modality to the other, and the percent of successful follow-up.

## Materials and Methods

The protocol for this trial and supporting CONSORT checklist are available as supporting information; see Checklist S1 and Protocol S1.

### Ethics statement

This study was conducted according to the principles expressed in the Declaration of Helsinki. Four Canadian tertiary-care teaching hospitals participated in this study, which was approved by the Research Ethics Boards at all of the centres (Hamilton Health Sciences, Ottawa Hospital, University Health Network, University of British Columbia-Vancouver Coastal Health Authority). All patients provided written informed consent.

### Eligibility criteria

Patients were eligible to participate in this study if they were at least 45 years of age, had an expected hospital length of stay of at least 48 hours, were undergoing a non-cardiothoracic surgical procedure that was amenable to neuraxial blockade, and met at least one of six criteria predictive of moderate- to high-risk for perioperative cardiorespiratory events (Tables 1 and 2). Patients were excluded from participation if they had a contraindication to neuraxial blockade, a prior adverse reaction to local anesthetics or opioids, previous coronary artery bypass graft surgery with complete revascularization in the preceding five years and no subsequent evidence of cardiac ischemia, or a history of pneumonia within two weeks of surgery. We also excluded patients who were already intubated or mechanically ventilated or had a concomitant life-threatening disease that was likely to limit their lifespan to less than 30 days.

**Table 1:** Inclusion criteria for the PeriOperative Epidural Trial Pilot Study.

Any patient undergoing non-cardiothoracic surgery and
1. is ≥45 years old;
2. has an expected length of stay ≥48 h;
3. is undergoing a procedure amenable to postoperative epidural analgesia; AND
4. fulfills any of the following SIX criteria
i. history of coronary artery disease;
ii. history of peripheral vascular disease;
iii. history of atherothrombotic stroke;
iv. hospitalization for congestive heart failure within three years of randomization;
v. undergoing major vascular surgery; OR
vi. has at least THREE of the following factors
a. any history of congestive heart failure,
b. diabetes currently requiring oral hypoglycemic or insulin therapy,
c. history of transient ischemic attack,
d. history of chronic obstructive pulmonary disease,
e. preoperative serum creatinine >175 µmol/L,
f. age >70 years,
g. anticipated duration of anesthesia ≥3 h,
h. intraperitoneal or intrathoracic surgery,
i. surgery that must be undertaken within 24 h of acute presentation to hospital

**Table 2:** Exclusion criteria for the PeriOperative Epidural Trial Pilot Study.

1. contraindication to epidural analgesia
i. stable platelet count <50,000 mm^−3^ or a falling platelet count <100,000 mm^−3^;
ii. abnormal INR or aPTT;
iii. ongoing use or planned peri-operative use of anticoagulants;
iv. systemic infection with elevated white blood cell count and temperature >37.5 degrees Celsius;
v. local infection at proposed site for epidural insertion;
vi. severe cardiac valvular abnormalities that do not tolerate afterload reduction;
vii. vertebral abnormalities that prevent proper placement of an epidural catheter or spread of epidural drugs;
2. prior adverse reaction to local anesthetics or narcotics;
3. previous coronary artery bypass graft surgery with complete revascularization in the preceding five years AND no evidence of cardiac ischemia since the procedure;
4. pneumonia within two weeks of surgery;
5. currently intubated or mechanically ventilated; OR
6. concomitant life-threatening disease likely to limit life expectancy to <30 days.

Patients were informed about the study during their visit to the centres' preoperative assessment clinics. Study coordinators kept a log of all eligible patients, the eligible patients who were not recruited and the reasons for their lack of participation, and the patients who were successfully recruited into the study. Study coordinators obtained informed consent from each subject; whenever possible, subjects had at least 24 hours to consider their participation.

### Randomization

After informed consent, subjects were randomly allocated to receive neuraxial anesthesia with or without general anesthesia and postoperative epidural analgesia (epidural group) or general anesthesia and postoperative i.v. opioid analgesia (IV group). We stratified the randomization by centre and by type of surgery (lower limb surgery *vs.* non-lower limb surgery) on the basis that epidural catheters would be sited in the lumbar and thoracic levels respectively in the epidural group. One member of the central coordinating office, who was not involved with any of the participating centres, generated the allocation sequence in random permuted blocks by computer; the sequence was stored on a secure SQL Server database in the coordinating office. At each centre, a research assistant obtained the allocation for a subject by logging onto a secure study website maintained by the central coordinating office. Allocation took place one to two hours before surgery to reduce the risk of including subjects who did not receive their scheduled surgery. The allocation was printed and placed in an opaque envelope, which was delivered to the anesthesiologist when the subject entered the operating room.

### Epidural group

In the epidural group, anesthesia was achieved by neuraxial blockade (epidural or combined spinal-epidural) with or without sedation or by a combination of neuraxial blockade and general anesthesia depending on the surgical procedure and the preferences of the subject and clinicians. All subjects had an epidural catheter inserted in the operating room prior to the start of surgery. The catheters were sited between the T4 and L5 vertebral levels, depending on the dermatomal levels that would be affected by the surgery; thus, catheters were sited in the lumbar region for lower limb procedures and in the thoracic region for all other surgical procedures. Whenever possible, the catheter was inserted at the vertebral interspace corresponding to the midpoint of the dermatomal range affected by the surgery. Proper placement of the epidural catheter was ascertained using a 3-mL test dose of lidocaine 1% to 2% with epinephrine 1∶200000. Intraoperative neuraxial blockade was achieved using long-acting local anesthetic (bupivacaine or ropivacaine).

Postoperative epidural analgesia was started upon emergence from general anesthesia or upon the return of sensation after neuraxial blockade. A combination of a long-acting local anesthetic (bupivacaine or ropivacaine) and an opioid (fentanyl, morphine, or hydromorphone) was used for the epidural infusion although the combinations varied between centres.

### IV group

In the IV group, anesthesia was achieved using a balanced general anesthetic. Postoperative analgesia was initiated upon emergence from general anesthesia and maintained using IV opioids (fentanyl, meperidine, morphine, or hydromorphone) administered as patient-controlled analgesia (PCA) and/or continuous infusion depending on the postoperative status of the subject.

### Clinical management

The management of anesthesia and postoperative analgesia was left to the attending anesthesiologists in the operating room and on the acute pain service. Postoperatively, the goal was to achieve a state of no or minimal pain (0 to 3 out of 10 on a numeric rating scale) in the subjects with minimal side effects. Acetaminophen and non-steroidal anti-inflammatory agents were permitted as co-analgesics. Epidural and i.v. opioid analgesia were maintained until the subjects were able to tolerate oral intake and analgesia was easily achieved with oral analgesics. All other clinical decisions related to subjects were made by the subjects' surgical team.

### Blinding

Investigators, who were not involved in the care of the subjects, obtained the bedside data (*e.g.*, clinical signs from the respiratory examination). In order to blind the investigators, subjects in the IV group had an epidural catheter (“sham epidural”) taped to the surface of their backs prior to induction of anesthesia. The epidural and intravenous tubings and the analgesic delivery system were covered during the bedside examinations. An unblinded research assistant collected data from the subject's medication record and chart and entered all of the data to avoid unblinding the investigator. An independent, blinded committee adjudicated any potential clinical events. The database staff and the data analysts were not blinded to the allocation.

### Outcomes

All outcome measures were defined *a priori*. Our primary outcome measures for this pilot study were the recruitment rate, the percent of eligible patients recruited, the crossover rate, and the percent of successful follow-up at the participating centres. The centre's recruitment rate was defined as the number of eligible patients who participated at the centre over the duration of the centre's recruitment period and was expressed as subjects per week. The study recruitment rate was defined as the mean of all centres' recruitment rates and the standard error of the mean. The percent of all eligible patients who participated was also calculated. We expressed follow-up as the percent of randomized subjects who were successfully followed for the 30-day study period. Similarly, we expressed crossover as the percent of subjects, receiving the allocated intervention, who switched to the alternate intervention. The percent crossover was calculated separately for each group.

We considered recruitment feasible for a large multicentre RCT if we were able to recruit one subject per centre per week (*i.e.*, 200 subjects from four centres over 50 weeks). We considered the pilot study to be successful, and a large multicentre RCT to be feasible, if we were able to recruit at least 70% of all eligible patients, no more than 5% of all recruited subjects crossed over from one modality to the other, and we achieved complete follow-up in at least 95% of all recruited subjects.

The secondary outcome measures were the clinical outcome measures that would be used if a large multicentre RCT was feasible. The detailed definitions for these outcomes are summarized in Tables 3 and 4. The primary clinical outcome, which would be used in a full-scale RCT, was a combined 30-day outcome of all-cause mortality, nonfatal myocardial infarction, cardiac arrest, postoperative pneumonia, and respiratory failure. Other secondary outcomes included thromboembolic events (deep vein thrombosis, pulmonary embolism, transient ischemic attack, stroke) and congestive heart failure during the first 30 postoperative days, and clinically significant bradycardia and clinically significant hypotension during the period in which postoperative epidural or IV opioid analgesia was used.

**Table 3:** Definition of primary clinical study outcomes to be used if a large multicentre trial is conducted.

**Primary Clinical Outcome** – Combined 30-day outcome of:	
Death	Any death regardless of cause
Nonfatal myocardial infarction	A typical rise of troponin OR a typical fall of an elevated troponin OR a rapid rise and fall of CK-MB and one of the following:
	1) characteristic ischemic symptoms;
	2) development of pathological Q waves on electrocardiogram;
	3) electrocardiographic changes indicative of ischemia;
	4) coronary artery intervention; OR
	5) new or presumed new cardiac wall motion abnormality on echocardiographic or radionuclide imaging
	OR pathological findings of acute myocardial infarction
Cardiac arrest	Any successful resuscitation from a documented or presumed ventricular fibrillation OR sustained ventricular tachycardia OR asystole
Clinically significant postoperative pneumonia	Any condition with documented hypoxemia (PaO_2_/FiO_2_ ratio ≤250) or fever (temperature >37.5 degrees Celsius) that meets either of the following criteria:
	1) rales or dullness to percussion on chest examination AND any of a) new onset of purulent sputum or change in sputum character, OR b) organism isolated from blood culture, OR c) pathogen isolated from transtracheal aspirate, bronchial brushing, or biopsy; OR
	2) new or progressive infiltrate, consolidation, cavitation, or pleural effusion on chest radiograph AND any of a) criteria a, b, or c, as above OR b) isolation of virus or detection of viral antigen in respiratory secretions, OR c) diagnostic antibody titers, OR histopathologic evidence of pneumonia
Respiratory failure	Any condition requiring intubation of the trachea and mechanical ventilation AFTER completion of surgery, emergence from anesthesia, successful extubation (if intubated during surgery), and spontaneous ventilation for ≥1 h after surgery

**Table 4:** Definition of secondary clinical study outcomes to be used if a large multicentre trial is conducted.

Deep vein thrombosis	Any clinical suspicion of deep vein thrombosis (lower limb pain OR tenderness OR swelling OR edema) AND objective diagnostic confirmation (positive lower limb venogram with constant intraluminal filling defect seen on ≥2 views OR compression ultrasound demonstrating a noncompressible vein segment)
Pulmonary embolus	Any clinical suspicion of pulmonary embolus (chest pain OR shortness of breath) AND objective diagnostic confirmation
	1) definite pulmonary embolus = a pulmonary angiogram with a constant intraluminal filling defect OR a spiral computed tomogram with an unenhanced filling defect seen in a central pulmonary artery OR a high-probability ventilation-perfusion scan OR an intermediate ventilation-perfusion scan with venographic evidence of deep vein thrombosis OR autopsy evidence of pulmonary embolus
	2) probable pulmonary embolus = an intermediate ventilation-perfusion scan with clinical signs
Transient ischemic attack	Any new focal neurological deficit of vascular origin that lasts <24 h with no permanent neurological sequelae
Stroke	Any new focal neurological deficit of vascular origin with signs and symptoms lasting ≥24 h
Congestive heart failure	Any condition with both clinical (elevated jugular venous pressure OR respiratory rales OR crepitations OR presence of S3) AND radiological (vascular redistribution OR interstitial pulmonary edema OR frank pulmonary edema) evidence consistent with congestive heart failure
Clinically important bradycardia	Heart rate <60 bpm requiring temporary pacing, a sympathomimetic agent, or atropine
Clinically important hypotension	Systolic blood pressure that is at least 20% lower than the preoperative systolic blood pressure AND requires fluid resuscitation, a vasopressor, or an inotropic agent

### Data collection

We collected demographic data when informed consent was obtained. The subject's physical status and levels of cardiovascular and respiratory risk were described using the American Society of Anesthesiologists physical status classification [23], the Revised Cardiac Risk Index [4], [24], and the Postoperative Pneumonia Risk Index [3] respectively. Details on the anesthetic and postoperative analgesic were collected immediately after surgery and then daily while the subjects were receiving epidural or IV opioid analgesia. Postoperative pain intensity, at rest (static pain) and with movement (dynamic pain), were measured using a 0 to 10 numeric rating scale (NRS; 0 = no pain, 10 = worst possible pain) every four hours after surgery while the subjects were receiving epidural or IV narcotic analgesia.

For the clinical outcomes, we recorded an electrocardiogram (ECG) six to 12 hours postoperatively and on the first, second, and 30^th^ day after surgery. We obtained troponin I levels 6 to 12 hours postoperatively and on the first, second, and third day after surgery. If there was clinical suspicion of pneumonia (*i.e.*, new cough, sputum, dyspnea, fever, altered mental status, or abnormalities of the white blood cell count or arterial blood gas), we obtained a chest radiograph and a blinded investigator performed a pulmonary examination. Additional tests were ordered at the discretion of the subjects' clinicians. The subjects' charts were reviewed for any outcomes prior to hospital discharge. Thirty days after surgery, a research coordinator interviewed subjects by telephone. If subjects indicated they had experienced an outcome within the first 30 days after surgery, their physicians were contacted to acquire the appropriate documentation.

### Data management

For data entry, we used secure, web-based, electronic case report forms developed by the central coordinating office. Data were entered online daily during each subject's hospital stay and were stored in a secure SQL Server database. Source documents were stored locally in locked premises at each centre. All computer systems used in the study were located in locked, high-security areas of the Centre for Clinical Epidemiology and Evaluation at the Vancouver Coastal Health Research Institute.

### Statistical methods

Preliminary sample size calculations prior to this pilot study suggested the need for a sample size as low as 932 subjects (25% incidence rate, 30% relative risk reduction, 5% type I error rate, and 80% power) to as high as 8604 subjects (10% incidence rate, 20% relative risk reduction, 5% type I error rate, and 90% power) for a full-scale multicentre RCT. The need for a more precise estimate of the incidence rate was one reason to conduct a pilot study. For this pilot study, we based our sample size on the number of participating centres and the minimum weekly recruitment per centre that we would tolerate if the true incidence rate was 20% (yielding a sample size of 1250 for a 25% relative risk reduction, 5% type I error rate, and 90% power). Our experience with the PeriOperative ISchemic Evaluation (POISE) Trial [13] indicated that 25 centres would likely participate in Canada. To ensure that the results of a large RCT would be clinically relevant (*i.e.*, not outdated with respect to clinical management) at least one subject should be recruited weekly from each centre (1250 subjects/25 sites/50 subjects per site per year = 2 years). Thus, our target sample size was 200 subjects (one subject per week per centre for 50 weeks for 4 centres).

Continuous data were summarized with means and standard deviations for normally distributed data and with medians and interquartile ranges for skewed data. Discrete data were reported as absolute numbers and percentages. We anticipated that the number of pain score measurements over each day could vary between subjects; therefore, pain score data for each group and time interval were summarized using means and standard deviations weighted by the number of measurements per subject [25]. We reported the number of events in each group but we did not make any comparisons using inferential statistics as this pilot study did not have the power to detect meaningful differences. For the primary clinical outcome that would be used in a full-scale RCT, the event rate and its 95% confidence interval (CI) were calculated for each group. Data from all subjects were reported as randomized in keeping with the intention-to-treat principle.

## Results

**Figure 1:**
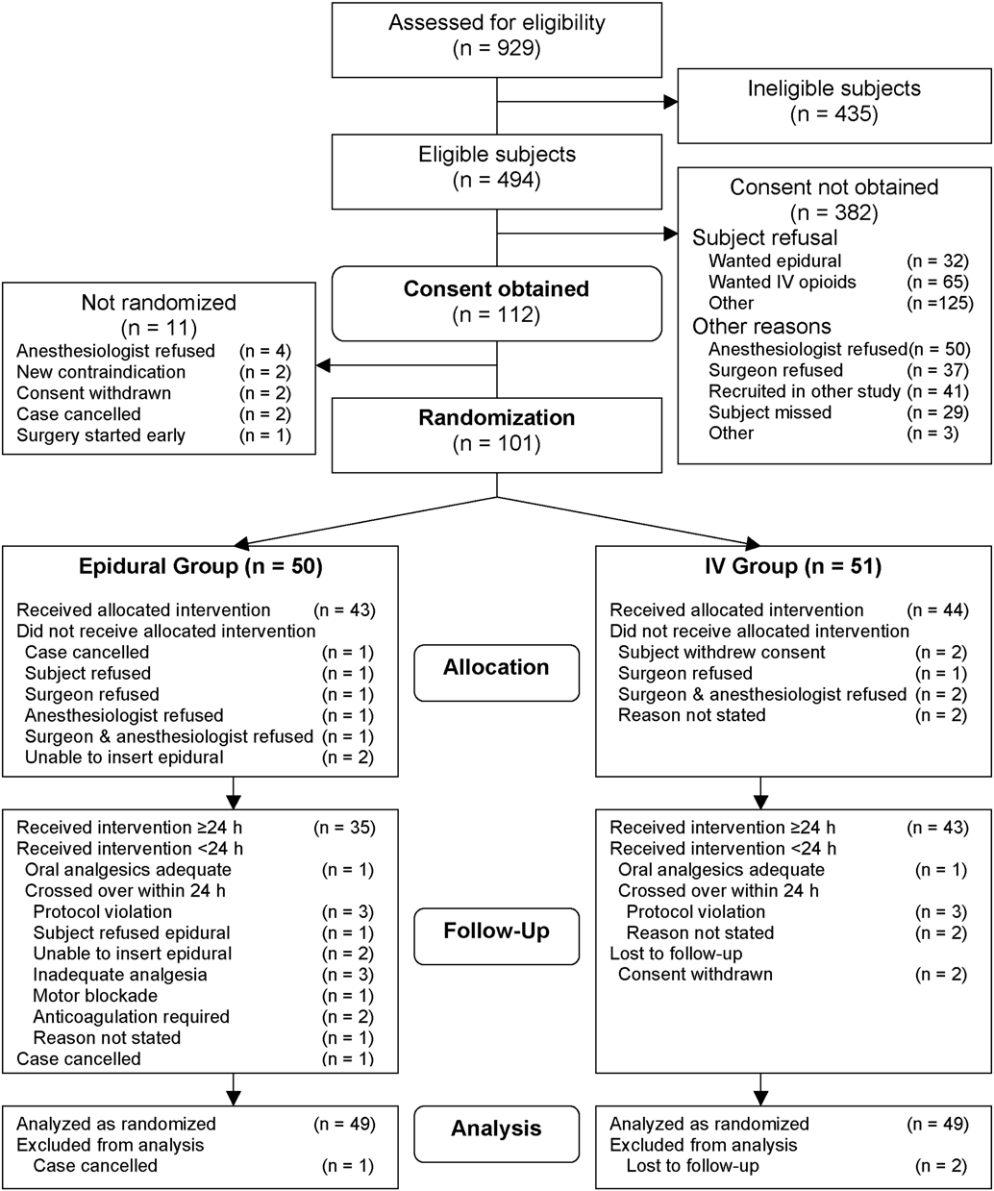
Flow of subjects through the PeriOperative Epidural Trial Pilot Study.

Figure 1 describes the flow of subjects in the POET Pilot Study. From May 25, 2005 to August 31, 2006, 929 patients were screened in the preoperative assessment clinics at the Hamilton Health Sciences (McMaster Site), Ottawa Hospital (Civic Campus), Toronto General Hospital, and Vancouver General Hospital. The study recruitment rate was 0.5±0.3 subjects per week per centre; the recruitment rate varied between centres (Table 5). Four hundred and ninety-four patients were eligible; 112 (22.7%) consented to participate in this study. Of the 112 consenting subjects, 101 were successfully allocated – 50 to the epidural group and 51 to the IV group. Eleven subjects could not be randomized for various reasons (Figure 1). Thirteen of the 49 subjects (26.5%) in the epidural group had crossed over to i.v. opioid analgesia due to refusal of the subject to receive an epidural catheter (*n* = 1), refusal of the clinicians (*n* = 3), failure to insert the epidural catheter (*n* = 2), inadequate analgesia (*n* = 3), excessive motor blockade (*n* = 1), development of postoperative complications that required treatment with anticoagulants (*n* = 2), or unstated reasons (*n* = 1). Five subjects (9.8%) in the IV group crossed over to epidural analgesia due to refusal of the clinicians (*n* = 3) or unstated reasons (*n* = 3); none were due to inadequate analgesia or complications related to IV opioids. Another three subjects never received the allocated intervention due to withdrawal of consent (*n* = 2) or cancellation of surgery (*n* = 1). By the end of the first 24 hours after surgery, 35/50 (70.0%) and 43/51 (84.3%) of the subjects in the epidural and IV groups respectively were receiving their allocated interventions. Ninety-eight of the 101 (97.0%) randomized subjects were followed for the entire study period.

**Table 5:** Recruitment rates of participating centres in the POET Pilot Study.

Centre	Eligible Patients, *n*	Consented Subjects, *n (%)* *	Weeks of Recruitment	Recruitment Rate, *subject/week (95% CI)*
Hospital A	117	35 (30)	50	0.7 (0.6–0.8)
Hospital B	31	10 (32)	26	0.4 (0.2–0.6)
Hospital C	206	57 (28)	64	0.9 (0.8–1.0)
Hospital D	140	10 (7)	56	0.2 (0.1–0.3)

Abbreviation: 95% CI, 95% confidence interval.

* Percentage denotes the percentage of eligible patients who consented to participate.

Tables 6 and 7 report the characteristics of the subjects, surgery, and anesthetic in each group.

**Table 6:** Characteristics of subjects recruited in the PeriOperative Epidural Trial Pilot Study.

Descriptor	Epidural Group (*n* = 49)	IV Group (*n* = 49)
**Mean age±SD**, *year*	70.9±7.6	70.3±8.6
**Sex**, *n (%)*		
Female	12 (24.5)	15 (30.6)
Male	38 (77.6)	36 (73.5)
**Comorbid conditions**, *n (%)*		
Coronary artery disease	14 (28.6)	12 (24.5)
Peripheral vascular disease	13 (26.5)	11 (22.4)
Stroke/transient ischemic attack	1 (2.0)	4 (8.2)
Congestive heart failure	2 (4.1)	0 (0.0)
Hypertension	34 (69.4)	31 (63.3)
Diabetes mellitus	11 (22.4)	14 (28.6)
Chronic renal dysfunction	2 (4.1)	0 (0.0)
Smoker		
Former	18 (36.7)	24 (49.0)
Current	11 (22.4)	7 (14.3)
Chronic obstructive pulmonary disease	4 (8.2)	7 (14.3)
**Medication history**, *n (%)*		
Aspirin	16 (32.6)	21 (42.8)
Clopidogrel/ticlopidine	2 (4.1)	2 (4.1)
Warfarin	1 (2.0)	2 (4.1)
Heparins	1 (2.0)	2 (4.1)
Statins	26 (53.1)	22 (44.9)
Nitrates	2 (4.1)	4 (8.2)
Diuretics	15 (30.6)	15 (30.6)
ACE inhibitors/angiotensin II receptor blockers	23 (46.9)	19 (38.8)
Beta-adrenergic blockers	16 (32.6)	21 (42.8)
Calcium channel blockers	8 (16.3)	17 (34.7)
Digoxin	2 (4.1)	3 (6.1)
Amiodarone	1 (2.0)	2 (4.1)
Beta-agonist bronchodilators	1 (2.0)	1 (2.0)
Inhaled steroids	3 (6.1)	1 (2.0)

Abbreviation: ACE, angiotensin converting enzyme; SD, standard deviation.

**Table 7:** Level of risk and type of surgery and anesthesia by group.

Descriptor	Epidural Group (*n* = 49)	IV Group (*n* = 49)
**Risk scores**		
ASA physical status, *n (%)*		
Class I	0 (0.0)	0 (0.0)
Class II	16 (32.6)	13 (26.5)
Class III	28 (57.1)	30 (61.2)
Class IV	4 (8.2)	5 (10.2)
RCRI score, *n (%)*		
0 risk factor	3 (6.1)	3 (6.1)
1 risk factor	23 (46.9)	25 (51.0)
2 risk factors	20 (40.8)	15 (30.6)
≥3 risk factors	3 (6.1)	6 (12.2)
PPRI score, *n (%)*		
Class 1 (0–15 points)	5 (10.2)	4 (8.2)
Class 2 (16–25 points)	19 (38.8)	18 (36.7)
Class 3 (26–40 points)	22 (44.9)	25 (51.0)
Class 4 (41–55 points)	2 (4.1)	1 (2.0)
Class 5 (>55 points)	0 (0.0)	0 (0.0)
**Type of surgery**, *n (%)*		
Intra-abdominal	28 (57.1)	26 (53.1)
Gynecological	3 (6.1)	6 (12.2)
Urological	6 (12.2)	7 (14.3)
Vascular	13 (26.5)	11 (22.4)
**Type of anesthesia**		
General anesthesia only	6 (12.2)	44 (89.8)
GA+neuraxial blockade		
GA+thoracic epidural	32 (65.3)	3 (6.1)
GA+lumbar epidural	5 (10.2)	1 (2.0)
GA+spinal	1 (2.0)	0 (0.0)
GA+combined spinal-epidural	0 (0.0)	0 (0.0)
Neuraxial anesthesia only		
Thoracic epidural	1 (2.0)	1 (2.0)
Lumbar epidural	4 (8.2)	0 (0.0)
Spinal	0 (0.0)	0 (0.0)
Combined spinal-epidural	0 (0.0)	0 (0.0)

Abbreviation: ASA, American Society of Anesthesiologists; GA, general anesthesia; PPRI, Postoperative Pneumonia Risk Index; RCRI, Revised Cardiac Risk Index.

**Figure 2:**
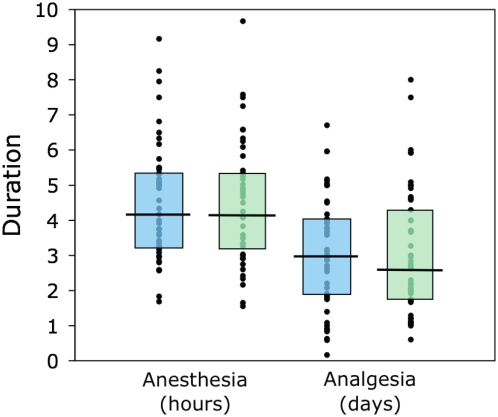
Box plots of duration of intraoperative anesthesia in hours and postoperative analgesia in days. Blue denotes the epidural group; green denotes the IV group. Circles indicate individual data points, the horizontal bars denote the median durations, and the boxes denote the interquartile range.

**Figure 3:**
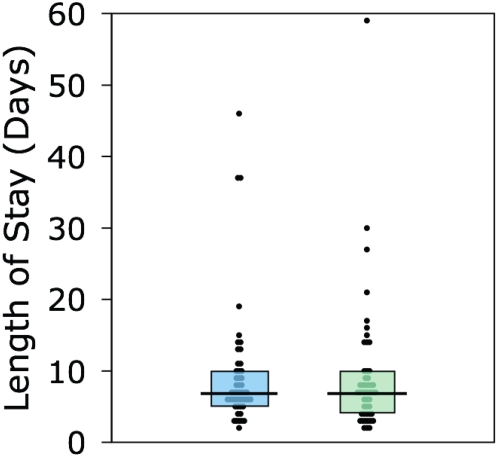
Box plot of duration of hospital stay in days. Blue denotes the epidural group; green denotes the IV group. Circles indicate individual data points, the horizontal bars denote the median durations, and the boxes denote the interquartile range.

Figure 2 and 3 summarizes the duration of anesthesia, postoperative analgesia, and length of hospital stay in each group. The central tendency and dispersion of the data were similar in both groups for all three time periods. Subjects in the epidural group received epidural or IV opioid analgesia for a median of 3.0 days (IQR 1.9–4.0 days). Those in the IV group received IV opioid or epidural analgesia for a median of 2.7 days (IQR 1.8–4.3 days). When the data were summarized on the basis of the actual analgesic received, subjects received epidural and IV opioid analgesia for a median of 3.7 days (IQR 2.4–4.5 days) and 2.4 days (IQR 1.7–3.7 days) respectively. The median length of hospital stay was 7 days in both groups and all reported clinical events were observed during that period.

Table 8 reports the clinical events observed in this study. The event rate for the combined outcome of all-cause mortality, non-fatal myocardial infarction, non-fatal cardiac arrest, postoperative pneumonia, and respiratory failure was 16.3% (8/49; 95% CI 8.5%–29.0%) in the epidural group and 14.2% (7/49; 95% CI 7.1%–27.0%) in the IV group. One other subject in the IV group died after the study period on the sixtieth day after surgery. Thromboembolic events (two deep vein thromboses, one pulmonary embolus, and one stroke) were observed in four subjects in the IV group. Two subjects in the epidural group had clinically significant bradycardia; three subjects (two in the epidural group, one in the IV group) had clinically significant hypotension; and one subject experienced a sudden postoperative loss of consciousness, which was attributed to the epidural analgesia. This subject did not suffer any sequelae from the serious adverse event. We have not made any between-group comparisons of the clinical events as this study was not powered for such comparisons and conclusions from such comparisons would be misleading.

**Table 8:** Number of events observed by clinical outcome and group.*

30-day Clinical Outcomes	Epidural Group (*n* = 49)	IV Group (*n* = 49)
**Primary outcome**, *n (%; 95% CI)*	8 (16.3; 8.5–29.0)	7 (14.2; 7.1–27.0)
**Outcomes comprising combined primary outcome**, *n (%)*		
Death	0 (0.0)	1 (2.0)†
Nonfatal myocardial infarction	4 (8.2)	2 (4.1)
Cardiac arrest	0 (0.0)	0 (0.0)
Postoperative pneumonia		
Definite	1 (2.0)	1 (2.0)
Probable	3 (6.1)	1 (2.0)
Respiratory failure	0 (0.0)	2 (4.1)
**Secondary outcomes**, *n (%)*		
Deep vein thrombosis	0 (0.0)	2 (4.1)
Pulmonary embolus		
Definite	0 (0.0)	1 (2.0)
Probable	0 (0.0)	0 (0.0)
Transient ischemic attack	0 (0.0)	0 (0.0)
Stroke	0 (0.0)	1 (2.0)
Congestive heart failure	0 (0.0)	0 (0.0)
Clinically important bradycardia	2 (4.1)	0 (0.0)
Clinically important hypotension	2 (4.1)	1 (2.0)
Serious adverse event		
Loss of consciousness	1 (2.0)	0 (0.0)

Abbreviation: 95% CI, 95% confidence interval.

* Note that zero events in a sample of 49 subjects still yields an upper 95% confidence limit of 0.073 for the 30-day incidence rate.

† In addition to the subject who died within the 30-day study period, another subject died 60 days after surgery.

**Figure 4:**
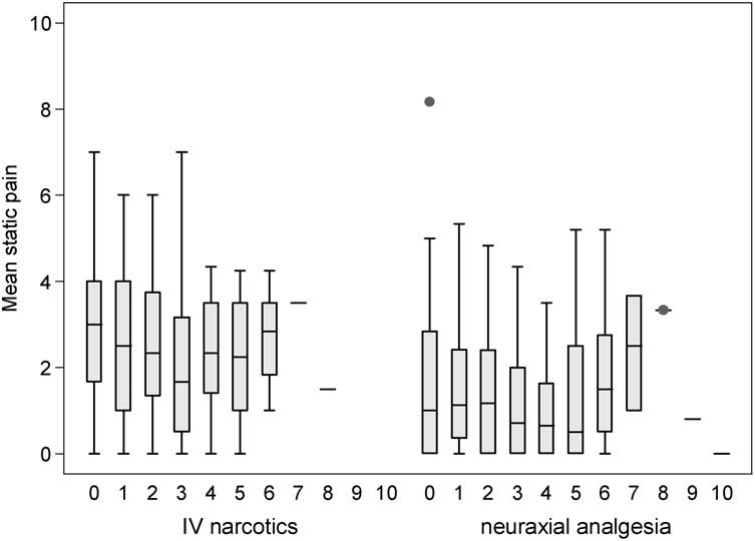
Box plots of numeric rating scale scores for static pain by group and postoperative day. The horizontal bars denote the median pain score, the boxes denote the interquartile range, the lower whiskers denote the lowest value lying within the first quartile subtract 1.5 times the interquartile range, the upper whiskers denote the highest value lying within the third quartile plus 1.5 times the interquartile range, and dots denote outliers.

Subjects in both groups received adequate analgesia to relieve static pain. Static pain scores over the duration of the intervention, expressed as weighted mean±standard deviation, were 1.5±1.2 in the epidural group and 2.5±1.3 in the IV group. Figure 4 depicts the pain scores by postoperative day for each group. Too few dynamic pain scores were recorded in subjects (epidural group, median 6 measurements; IV group, median 4 measurements) to permit meaningful summary or interpretation.

## Discussion

Over a 66-week period, the study recruitment rate was 0.5±0.3 subjects per week per centre, which was approximately half of the target recruitment rate of 1 subject per week per centre. Research staff from four Canadian tertiary-care teaching hospitals screened 929 patients and recruited 112 (22.7%) of 494 patients who were eligible for this pilot study. A total of 18 subjects (13 from the epidural group; 5 from the IV group) crossed over to the alternative group regardless of reason. The crossover rate in the epidural group due to failed or inadequate analgesia or complications from epidural analgesia was 14.3% (7/49), which exceeded the target crossover rate of no more than 5%. There were no crossovers in the IV group related to inadequate analgesia or complications from IV opioids. We achieved our target follow-up rate and successfully followed 97.0% (98/101) of the randomized subjects.

Our target recruitment rate was ambitious. In trials, published in this millennium, that compared epidural analgesia to IV analgesia, recruitment rates ranged from 0.5 to 3.5 subjects per *month* per centre (Table 9) [21], [26]–[30]. Although the recruitment rates were within the range reported in other studies, even in the centre with the lowest recruitment rate in our study, the percent of eligible patients recruited was low compared to other trials (Table 9).

**Table 9:** Rates of recruitment, crossover, and follow-up reported in recent randomized controlled trials evaluating epidural analgesia.

Study	Recruitment Period	Number of Subjects	Number of Centres	Recruitment Rate *Subject/month/centre*	Eligible Patients Recruited *n (%)*	Crossover *n (%)*		Complete Follow-up *n (%)*
						Epidural to IV	IV to Epidural	
Norris 2001 [21]	August 1993–July 1997	160	1	3.5	175/247 (70.8)	N/R	N/R	N/R
VACS 2001 [22]	N/R	1021	15	N/R	1021/1371 (74.5)	32/489 (6.5)	48/495 (9.7)	973/984 (98.9)
Steinberg 2002 [27]	July 1997–August 1998	48	5	0.7	N/R	N/R	N/R	41/48 (85.4)
Carli 2002 [28]	April 1998–April 2000	64	2	1.3	N/R	N/R	N/R	64/64 (100)
MASTER 2002 [21]	July 1995–May 2001	920	25	0.5	N/R	29/447 (6.5)	19/441 (4.3)	N/R
Katz 2003 [29]	August 1995–August 2000	212	2	1.8	212/684 (31.0)	N/A*	N/A*	N/R
Zutshi 2005 [30]	N/R	59	1	N/R	N/R	6/31 (19.4)	0/28 (0.0)	35/59 (59.3)
POET Pilot 2008	May 2005–August 2006	112	4	2.0	112/494 (22.7)	7/49 (14.3)	0/49 (0.0)	98/101 (97.0)

Abbreviations: N/A, not applicable; N/R, not reported.

* All patients received IV patient-controlled analgesia after surgery.

Our ability to recruit subjects was limited by several factors. First, nearly half (222/494; 44.9%) of all eligible subjects declined to participate (Figure 1). Ninety-seven (19.6%) of the eligible patients had decided on either epidural analgesia or intravenous PCA already by the time they were approached by the study coordinators. Another 125 (25.3%) declined for various reasons, of which the most common was a lack of interest or aversion, expressed by the patient or the family members, in being a research subject. We could not determine whether the comparison of an invasive intervention (epidural analgesia) to a relatively non-invasive intervention (IV analgesia) influenced the patients' decisions. However, our observations are consistent with those of other investigators, who have found epidural anesthesia to be a deterrent to participation in clinical research [31], [32].

Second, competition between another RCT affected our study's recruitment. All four centres were participating in the POISE Trial [14], a study with similar selection criteria and outcome measures [34], at the time of this study. We had hypothesized that concurrent conduct of the two similarly designed studies would increase efficiency in screening, consent, and follow-up. We anticipated that most moderate- to high-risk patients would be eligible for one of the two studies, but not both. As the POISE Trial had started before the POET Pilot Study, we had agreed *a priori* that any patient eligible for both studies would be invited to participate in the POISE Trial. This situation occurred in 41 (8.2%) of our eligible patients. We estimate that another 12 patients would have enrolled in our study in the absence of the POISE Trial.

Third, the comfort level of clinicians with leaving the choice of analgesia to chance may have affected our ability to recruit subjects at one of the four centres. At each centre, the members of the departments of anesthesia and surgery had agreed to participate in this study. The four centres had similar, well-established epidural and intravenous analgesic policies and active postoperative pain services, yet anesthesiologists and surgeons prevented 87 patients (17.6%) from participating (Figure 1). Seventy-seven (88.5%) of these cases involved vascular patients at one centre (Hospital D). At that centre, the standard postoperative analgesic modality was epidural analgesia for supraumbilical procedures and intravenous analgesia for infraumbilical procedures. Although clinicians were comfortable recruiting other types of moderate- to high-risk surgical patients at that centre, they were reluctant to change their practice regarding vascular patients.

The reluctance to leave the choice of analgesia to chance was also seen after consent and allocation (Figure 1). Anesthesiologists prevented another four subjects from undergoing allocation and surgeons and anesthesiologists refused to use the allocated intervention (*i.e.*, protocol violation) in six subjects. In these ten cases (Hospital A *n* = 2; Hospital C *n* = 6; Hospital D *n* = 2), the clinicians decided that one of the two analgesic modalities would be of greater benefit for their particular subject and were unwilling to accept the alternative. Clinicians cited their own expert knowledge, concerns of patient safety, fears of inadequate analgesia (when allocation was to the IV group), or too much analgesia (when allocation was to the epidural group) as reasons for preventing allocation or violating the protocol.

Failed or inadequate analgesia or complications from epidural analgesia led to discontinuation of epidural analgesia in seven subjects in the epidural group (Hospital A *n* = 1; Hospital B *n* = 2; Hospital C *n* = 3; Hospital D *n* = 1). The number of cases at each centre was too small to determine whether variations in clinical practice between centres played any role. The crossover rate from epidural to IV analgesia in our study (14.3%) was in keeping with rates reported in recent RCTs, which have ranged from 6.5% to 19.4% with larger trials reporting lower rates of crossover (Table 9) [21], [22], [30].

Our original intent was to conduct a national multicentre RCT over a two-year period in approximately 25 centres. Based on the results of this pilot study, our original plan is infeasible. Using the lower 95% confidence limit for the event rate in the IV group (7.1%), 25% relative risk reduction, 5% type I error, and 80% power, the sample size would be 6148. Sixty-two centres would need to recruit 0.5 subjects per week to complete recruitment in four years. A study of that size and duration would need to be international and would be very expensive.

We conducted this study in academic centres of two Canadian provinces only. Preliminary work using the Canadian Institute for Health Information Discharge Abstract Database indicated a wide variation in the use of neuraxial blockade between provinces and between academic and community hospitals in Canada. For example, the rate of neuraxial blockade for major joint arthroplasties in Canada during the year 2000 varied from 39.9% (British Columbia) to 65.6% (Alberta) for academic centres and from 19.2% (Alberta) to 33.0% (Ontario) for community hospitals [Unpublished work by Peter Choi, P.J. Devereaux, Gordon Guyatt, and Bruce Weaver]. Thus, our results may not reflect the research culture or the feasibility of conducting an RCT comparing epidural analgesia to IV analgesia in centres from other Canadian provinces or countries or in community hospitals in British Columbia and Ontario.

In summary, the results of this pilot feasibility RCT suggest that a large, multicentre RCT is an impractical design for comparing epidural analgesia with IV analgesia with regards to perioperative cardiorespiratory outcomes. Our results show patients and clinicians are reluctant to leave the choice of postoperative analgesia to chance. Several other barriers make a large RCT impractical. Recent work suggests changes in perioperative care over the past few decades are resulting in reductions in perioperative complications. Pöpping and colleagues [34] show decreases over the past 35 years in the risk of postoperative pneumonia with systemic analgesia, which has lessened the potential benefits of epidural analgesia and would necessitate larger sample sizes to detect clinically relevant and plausible differences in outcomes between analgesic modalities. The use of epidural analgesia across diverse surgical populations raises the issue of generalizability of results *versus* applicability of results to a specific population. Whereas the inclusion of many types of patients will increase the generalizability of the results of an RCT if a difference is found, clinical heterogeneity will be criticized if an RCT fails to detect a difference. Clinicians will likely argue that the wrong population was studied in the latter case; however, restriction to a specific population can increase the difficulty in recruitment due to a lower number of eligible patients. Lastly, the costs and labour associated with RCTs are high relative to observational studies.

Considering the challenges of using RCT methods to study the effect of epidural blockade on clinical outcomes, observational methods, such as a prospective cohort study, may be more feasible though subject to more biases. Furthermore, research using qualitative methods, is needed to explore the barriers to the conduct of randomized controlled trials comparing neuraxial blockade to non-invasive interventions [35].

## Supporting Information

Protocol S1 Trial Protocol
(1.23 MB PDF)

Checklist S1 CONSORT checklist
(0.06 MB DOC)

Contributions S1 Contributions of participants in the POET Pilot Study
(0.04 MB DOC)
