# Supplementary material for: Effects of Neuraxial Blockade May Be Difficult To Study Using Large Randomized Controlled Trials: The PeriOperative Epidural Trial (POET) Pilot Study
Source: PLoS One. 2009 Feb 27;4(2):e4644. doi: 10.1371/journal.pone.0004644 (PMC2645707; doi:10.1371/journal.pone.0004644)

The PeriOperative Epidural Trial (POET) Pilot Study

A small, pilot study of intraoperative spinal or epidural anesthesia and postoperative epidural analgesia versus general anesthesia and intravenous analgesia in patients undergoing non-cardiopulmonary surgery at moderate to high risk of perioperative cardiorespiratory events

Principal Investigators:

Dr. Peter Tsz-Lung Choi
UBC Department of Anesthesia /
Vancouver Coastal Health Research Institute

Dr. W. Scott Beattie
University of Toronto Department of Anesthesia

Co-Investigators:

McMaster University
Dr. Norman Buckley
Dr. P.J. Devereaux
Dr. James Paul
Dr. Corey Sawchuk

University of British Columbia
Dr. Keith Chambers
Dr. Mark Fitzgerald
Dr. York Hsiang
Dr. Hamed Umedaly

University of Ottawa
Dr. Ashraf Fayad
Dr. Homer Yang

University of Toronto
Dr. Vincent Chan

Central Coordinating Office:

Centre for Clinical Epidemiology and Evaluation
Vancouver Coastal Health Research Institute
828 West 10th Avenue
Vancouver, British Columbia
V5Z 1L8

This protocol has been developed by the POET Investigators and its content is the intellectual property of this group. It is an offence to reproduce or use the information or data in this protocol for any purpose other than the POET Pilot Study without prior approval from the authors.

Summary of Research Proposal

The **objectives of the POET Pilot Study** are to determine:
1) the feasibility of recruiting patients in a timely manner,
2) the surgical populations in which patients, anesthesiologists, and surgeons are willing to participate in a perioperative epidural trial, and
3) the rates of crossover from epidural analgesia to intravenous narcotic analgesia and *vice versa*.

Study Design	Multicentre (five hospitals) randomised controlled, investigator-blinded pilot study with 30-day follow-up
Sample Size	Two hundred and fifty patients (50 patients per site)
Selection Criteria	Any patient undergoing non-cardiopulmonary surgery is *eligible* if s/he: 1) is = 45 years old; 2) has an expected length of stay = 48 h; 3) is under-going a procedure amenable to postoperative epidural analgesia; AND 4) fulfils any of six criteria for moderate to high cardiorespiratory risk.<br>A patient will be *ineligible* for this study if s/he 1) has a contraindication to epidural analgesia; 2) had a prior adverse reaction to local anesthetics or narcotics; 3) had coronary artery bypass graft surgery with complete revascularization in the preceding 5 years and has no evidence of cardiac ischemia since the procedure; 4) has pneumonia in the preoperative period; 5) is intubated or mechanically ventilated prior to surgery; OR 6) has concomitant life-threatening disease likely to limit life expectancy to <30 days.
Interventions	Intraoperative neuraxial (epidural or spinal) ± general anesthesia AND postoperative epidural analgesia *versus* intraoperative general anesthesia AND postoperative intravenous narcotic analgesia
Outcomes	*Primary combined outcome* of 30-day all-cause mortality, nonfatal myocardial infarction, cardiac arrest, postoperative pneumonia, and respiratory failure. *Secondary outcomes* of deep vein thrombosis, pulmonary embolism, transient ischemic attack, stroke, congestive heart failure; *safety outcomes* of clinically significant bradycardia and clinically significant hypotension.

Information from this pilot study will guide sample size calculation and fine-tune the protocol for a future large randomized controlled trial that is needed to answer the question:

“In patients with moderate or high risk for cardiorespiratory complications, who are undergoing non-cardiothoracic surgery, does the use of perioperative neuraxial blockade reduce perioperative mortality and cardio-respiratory events compared to intraoperative general anesthesia (without epidural or spinal anesthesia) AND postoperative intravenous narcotic analgesia?”

Research proposal

1. The Need for a Trial

1.1. What is the problem to be addressed?

Perioperative cardiorespiratory events are frequent complications of non-cardiac surgery and result in significant morbidity, mortality, and cost.[1-5] The costs from medical complications after surgery are anticipated to exceed the costs from complications in medical patients by 2019.[1] Over 65% of perioperative deaths result from a perioperative cardiorespiratory event.[1-3] Perioperative cardiac complications resulted in $20 billion in health care costs in the United States in 1990;[6] these costs have increased to $100 billion by 2000.[1] Postoperative pneumonia resulted in longer hospital stays, higher costs,[3-5] and had become the most common postoperative infection by the 1990s.[7] Depending on the type of surgery and the level of cardiac risk, rates of major cardiac events in non-cardiac surgery have varied from 2% to 19% in prospective studies (Figure 1).[8-33] Similarly, rates of postoperative pneumonia in non-cardiopulmonary surgery have varied from 1.5% to 37% (Figure 2),[2,4,5,11,34-57] demonstrating both the heterogeneity in patients undergoing surgery and the magnitude of the problem.

Randomised clinical trials (RCTs) have studied a number of perioperative cardiac interventions including ß-blockers,[58-69] calcium channel blockers,[69-81] nitrates,[80,82-85] platelet inhibitors,[86-92] and a_2-agonists.[93-101] Meta-analyses of these agents provide encouraging evidence that ß-blockers and a_2-agonists may reduce perioperative myocardial infarction (MI) and cardiac mortality (Table 1);[69,100,101] however, the treatment effects were almost entirely due to one or two small studies for each drug.[65,97,101] In the absence of these trials, the pooled treatment effects are smaller and the remaining trials lack the power to show significant effects. Thus, the results are hypothesis-generating but are insufficient to provide a definitive guide for clinical practice.

Interventions to reduce perioperative respiratory risk include smoking cessation,[102] lung expansion maneuvres,[103] selective nasogastric decompression,[104] selective digestive tract decontamination,[105-107] nutritional support,[108] and use of sucralfate for stress ulcer prophylaxis.[109] Most of these interventions[104-108] were studied in critically ill patients and the findings are not clearly generalisable to other postoperative patients. Meta-analyses demonstrate an overall decrease in postoperative respiratory complications with incentive spirometry or deep breathing exercises and lower rates of postoperative pneumonia with selective nasogastric decompression or selective digestive tract decontamination (Table 2), but the findings are based on few RCTs with small sample sizes, are highly susceptible to selection bias, and must be interpreted with caution.

Several large ß-blocker RCTs are underway.[110] Although these perioperative trials are important, they focus only on reduction of cardiac risk. Large RCTs of perioperative respiratory interventions are uncommon except in the critical care setting. Considering the high frequency of cardiorespiratory complications following non-cardiopulmonary surgery, and the volume of this surgery, we need to address prevention in the perioperative context. Ideally, interventions should reduce both cardiac and respiratory complications in a wide range of perioperative settings. We suggest that perioperative neuraxial blockade may well meet this criterion and, therefore, is an extremely promising intervention and an excellent candidate for a large, rigorous RCT.

Description of neuraxial anatomy and neuraxial blockade

Neuraxial blockade refers to nerve conduction blockade obtained at the epidural or spinal regions of the central nervous system (Figure 3). The epidural space is a fat-filled space external to the dura mater, which encloses the intrathecal space, including the cerebrospinal fluid and the

spinal cord. Since the late 1800's, both the epidural and intrathecal spaces have provided routes of administration for anesthetics (which eliminate sensation and may eliminate motor activity as well) and analgesics (which relieve pain). Neuraxial blockade is denoted as *neuraxial anesthesia* when afferent sensory and efferent motor fibres are blocked. Neuraxial blockade is denoted as *neuraxial analgesia* when only afferent sensory fibres are blocked.

Conventionally, anesthesiologists inject drugs into the epidural space via a small catheter inserted into the epidural region, at the vertebral level corresponding to the dermatome that is at the midpoint of the innervation for the surgical pain stimulus. For example, if the pain stimulus originates from T6 to T12, as in a large abdominal incision, the epidural would be sited between T8 and T9 or between T9 and T10. The standard of practice is to insert the epidural catheter prior to surgery regardless of whether planned use is in the intraoperative (epidural anesthesia) or postoperative (epidural analgesia) period.

Epidural anesthesia, usually obtained by large volumes of highly concentrated local anesthetics with or without narcotics, is used during surgery to obtain sensory and motor blockade with varying sympathetic blockade, depending on the vertebral level of the catheter. Due to the anatomy of the nervous system, thoracic epidurals will block the sympathetic nervous system to a greater extent than lumbar epidurals. As appropriate doses of epidural anesthesia do not result in unconsciousness, patients may receive sedation or general anesthesia in conjunction with epidural anesthesia depending on the nature and duration of the surgical procedure, the operative position of the patient, and the patient's level of anxiety.

Epidural analgesia in the postoperative period provides pain relief with minimal loss of touch sensation, joint-position sense, or motor strength. Continuous low-dose infusions of a mixture of low concentration local anesthetic and narcotic minimise the side effects associated with epidural analgesia (hypotension, bradycardia, numbness, and muscle weakness with local anesthetics; nausea, vomiting, pruritis, and respiratory depression with narcotics).

Spinal anesthesia is used in the intraoperative period only. Drugs are administered by inserting a small-diameter needle through the dura mater and injecting local anesthetic with or without narcotic into the cerebrospinal fluid. The choice of agents depends on the expected duration of surgery. Intrathecal agents are injected at the low lumbar interspaces to avoid injury to the spinal cord, which usually ends at L1 in adults. The dermatomal spread of the anesthetic and the extent of the sympathetic blockade depend on the density of the injected agents, relative to cerebrospinal fluid, and the position of the patient. Spinal anesthesia is achieved using a single drug bolus with supplemental epidural anesthesia or analgesia (depending on the time period). The choice between intraoperative epidural versus spinal anesthesia is influenced by the anticipated duration of surgery, the type of surgery, and the desired speed of onset of the blockade. Spinal analgesia in the postoperative period is achieved by adding long-acting narcotics to the drug bolus given during the induction of spinal anesthesia.

Beneficial biological effects of neuraxial blockade

Neuraxial blockade has potentially beneficial effects on the cardiovascular, hematological, and respiratory systems. The resultant changes in catecholamines, prothrombotic mediators, and respiratory function begin during surgery and continue postoperatively; thus, the ideal neuraxial blockade to reduce cardiovascular, thrombotic, and respiratory events consists of intraoperative and postoperative blockade.

Human observational studies and RCTs have revealed that neuraxial blockade decreases perioperative levels of epinephrine and norepinephrine,[112,113] selectively vasodilates stenotic coronary arteries,[114] and improves myocardial perfusion.[116] These effects are likely mediated by both a- and ß-adrenergic sympathetic blockade.[115] Perioperative epidural blockade may

attenuate the hyper-coagulability, seen in the postoperative period, by decreasing plasminogen activator inhibitor activity,[116] factor VIII activation,[117,118] and inhibition of fibrinolysis,[117] and by increasing the rate at which antithrombin III returns to normal levels.[119] These effects are likely mediated by neuroendocrine mechanisms.[118]

Postoperative epidural analgesia may improve respiratory function by attenuating the postoperative reduction in pulmonary functional residual capacity and subsequent respiratory complications that are induced by surgery, especially thoracic and upper abdominal procedures, and general anesthesia.[120,121] Epidural analgesia also improves diaphragmatic function by blocking the reflex inhibition of phrenic nerve activity that occurs after upper abdominal surgery.[122] The use of local anesthetics for epidural analgesia reduces the requirement for epidural or systemic narcotics in the postoperative period and may decrease the risk of postoperative sedation and hypoxemia.[122]

The physiological benefits achieved by neuraxial blockade should, in theory, reduce perioperative thrombotic (e.g. MI, deep vein thrombosis [DVT]) and respiratory (pneumonia, respiratory failure) events. This physiological rationale requires confirmation in clinical trials; however, to date all RCTs of neuraxial blockade have been underpowered to detect clinically relevant differences in these perioperative outcomes.

Two moderate size RCTs have been published to date. The Veterans Affairs Cooperative Study[124] and the MASTER Anaesthesia Trial[125] studied 1021 and 888 moderate- to high-risk patients undergoing major abdominal operations respectively (Tables 3 and 4). Both RCTs compared combined epidural and general anesthesia and postoperative epidural analgesia to general anesthesia and postoperative intravenous narcotic analgesia and observed trends toward increased 30-day all-cause mortality[124,125] and myocardial infarctions[125] in the neuraxial blockade groups (Table 3). There were no differences in major postoperative morbid events with the exception of reduced nonfatal MI in abdominal aortic surgery (OR 0.32; 95% CI 0.17-0.92)[124] and reduced respiratory failure (OR 0.70; 95% CI 0.52-0.95)[125] with neuraxial blockade. The negative results seen in these two studies may have been due to the use of narcotic *without* local anesthetic to achieve postoperative epidural analgesia in the Veterans Affairs Cooperative Study[124] and 50.3% (225 / 447) compliance with the epidural protocol in the MASTER Anaesthesia Trial.[125]

Several meta-analyses have evaluated the effect of neuraxial blockade on perioperative outcomes (Tables 3 and 4). Neuraxial anesthesia reduced 30-day all-cause postoperative mortality (OR 0.70; 95% CI 0.54-0.90), DVT (OR 0.56; 95% CI 0.43-0.72), pulmonary embolism (OR 0.45; 95% CI 0.29-0.69), pneumonia (OR 0.61; 95% CI 0.48-0.76), and respiratory depression (OR 0.41; 95% CI 0.23-0.73) and showed a trend toward reduced postoperative MI (OR 0.87; 95% CI 0.45-1.00).[126] There was no difference in all-cause mortality or MI between postoperative epidural analgesia and intravenous analgesia of =24 hour duration;[127] however, there was a statistically significant reduction for MI in patients receiving thoracic epidural analgesia (OR 0.43; 95% CI 0.19-0.97).[131] The observed treatment effect is less convincing with inclusion of more recent RCT data in the meta-analysis (Table 3).[128] Postoperative epidural analgesia with local anesthetics also reduced respiratory infections compared to systemic narcotics (relative risk [RR] 0.36; 95% CI 0.21-0.65).[129] In summary, meta-analyses show consistent trends toward reduction in all-cause mortality with neuraxial anesthesia and analgesia. Data are consistent with decreases in cardiovascular and pulmonary events contributing to reductions in all-cause mortality, with both intraoperative and postoperative blockade playing a role in reducing mortality.

1.2. What is the principal research question to be addressed?

Principal research question of the POET Pilot Study

The principal research question of the POET Pilot Study is: "In the current clinical setting, is a large multi-centre RCT comparing neuraxial anesthesia and analgesia to general anesthesia and intravenous narcotic analgesia in patients undergoing non-cardiothoracic surgery feasible?"

Principal research question of a large multicentre trial

The answer to the preliminary question above is needed before we can answer our principal research question: "In patients with moderate or high risk for cardiorespiratory complications, who are undergoing non-cardiothoracic surgery, does the use of intraoperative neuraxial anesthesia AND postoperative neuraxial analgesia reduce perioperative mortality and cardio-respiratory events compared to intraoperative general anesthesia (without epidural or spinal anesthesia) AND postoperative intravenous narcotic analgesia?"

1.3. Why is a trial needed now?

Why is a pilot study needed now?

The pilot study will refine our sample size calculations *and* determine the feasibility of conducting a large multi-centre RCT to answer our principal research question. First, we need to determine the sample size required to detect a clinically relevant difference. The results of the pilot study will provide data to refine our current estimates of the rates of perioperative cardiorespiratory complications (based on pooled data from prospective studies; Figures 1 and 2) and determine the proportion of patients who switch from epidural analgesia to narcotic analgesia and *vice versa* (crossover rates).

Second, we need to determine the feasibility of recruiting large numbers of patients in a timely fashion. Epidural RCTs performed in the last decade have enrolled 0.5 to 3.5 patients per month per site, based on studies that have reported their enrolment periods (Table 5). As the anticipated sample size of a large multicentre RCT will require over 2000 patients (see Section 2.10), an enrolment rate of at least 1 patient per week per site would be needed for such a study to be feasible. This pilot study will determine the enrolment rate.

Third, we need to determine the surgical populations in which patients, anesthesiologists, and surgeons are willing to participate in an RCT in which one option, neuraxial anesthesia and analgesia, requires an invasive procedure (insertion of an epidural or spinal needle) and the other option, general anesthesia and intravenous narcotic analgesia, does not. It is possible that the willingness of patients, anesthesiologists, and surgeons to participate will vary depending on the type of surgery to be performed. This information will help us improve the efficiency of recruitment efforts for a large multi-centre RCT and determine the generalisability of the results of such a study.

Why is a large multicentre trial needed?

The trends toward reduction in cardiorespiratory events with neuraxial blockade observed in meta-analyses and RCTs are encouraging, but the discrepancy between meta-analyses and moderate-size RCTs with regards to all-cause mortality raises doubts about the use of neuraxial blockade. Because of the susceptibility to publication bias, and previous instances in which subsequent trials contradicted results of meta-analyses of small trials, clinicians are legitimately sceptical about considering a meta-analysis of many small trials as providing definitive evidence. Unfortunately, no RCT, including the Veterans Affair Cooperative Trial[124] and the MASTER Trial,[125] has had sufficient power to provide definitive evidence of reduction or increase in the crucial combined endpoint of death and major cardiorespiratory events.

The clinical uncertainty is reflected in the variation across Canada in the use of neuraxial blockade. For example, use of intraoperative neuraxial anesthesia during hip and knee replacement operations in Canada during 2000 varied from 19.2% in community hospitals in Alberta to 65.6% in academic hospitals in Alberta (Table 6). Despite legitimate reservation and resulting variation in practice, if neuraxial blockade really does result in appreciable decreases in major cardiorespiratory events and all-cause mortality, it should become the standard for operative anesthesia and postoperative analgesia. Clearly, clinicians need a definitive trial to resolve the issue.

1.4. References to relevant systematic reviews

Please see *Beneficial Biological Effects of Neuraxial Blockade* in Section 1.1.

1.5. How will the results of this pilot study be used?

A large multicentre RCT would provide the best evidence needed to answer our primary research question. This pilot study will help us determine whether such a trial would be feasible or not. If a large definitive RCT is feasible, we would initiate such a trial involving as many Canadian centres as possible; if it is not feasible, we would try to answer our primary research question using a prospective observational study.

1.6. What are the risks to the safety of participants involved in the trial?

As this pilot study will compare two methods of providing anesthesia and analgesia that are part of standard clinical practice, the risks to the safety of participants in the trial will not differ from patients who are not participating in this study. That is, their risk will be the risks of any individual, with moderate to high risk for cardiorespiratory complications, undergoing major surgery. Jenkins and Baker have reviewed the risks associated with anesthesia and analgesia recently;[135] these risks are summarised in the consent form for this pilot study (Appendix).

2. The Proposed Trial

2.1. What is the proposed trial design?

This study is a pilot study of an investigator-blinded RCT of neuraxial anesthesia and postoperative epidural analgesia versus general anesthesia with postoperative intravenous narcotic analgesia in patients at moderate- to high-risk for cardiorespiratory events.

To enhance generalisability, we will include any patient who are at moderate to high risk for cardiorespiratory events and who would normally be eligible for epidural analgesia. One approach to conducting this trial is to be very specific about a whole variety of clinical policies, such as choice of drugs, drug doses, and rate of administration in both intervention and control groups, but this approach would be logistically more difficult, as we attempt to change clinicians' existing practice and one could question the generalisability of the approach to existing clinical practice.

Our approach, in contrast, is to provide a pragmatic assessment of the impact of neuraxial blockade on common, important outcomes. As all of the participating centres have similar approaches and management policies for the treatment and control interventions of interest, basing the choice of drugs, drug doses, and rate of administration in both intervention and control groups on existing clinical practice will still achieve similar anesthetic and analgesic effects between sites while increasing the generalisability of the results. Figure 4 illustrates the trial design.

2.2. What are the planned trial interventions?

Treatment protocol

Epidural analgesia: Patients allocated to the epidural analgesia group will have an epidural catheter inserted prior to the start of surgery. Catheters will be inserted by the staff anesthesiologist or a senior anesthesia resident and will be sited between T4 and L5, depending on the dermatomal levels that will be affected by the surgical procedure. That is, epidural catheters will be sited in the lumbar region ("lumbar epidural") for procedures involving the lower limbs; epidural catheters will be sited in the thoracic region ("thoracic epidural") for all other eligible procedures. To ensure that the catheter is sited in the epidural space, and not in the intrathecal space or in a blood vessel, the catheter placement will be tested using a small dose (~3 mL) of lidocaine 1%-2% with epinephrine 1:200,000. Absence of sensorimotor blockade and absence of changes in the cardiovascular or central nervous systems will confirm proper siting of the epidural catheter. Depending on the type of surgery, neuraxial blockade will be initiated and maintained in the intraoperative period with spinal OR epidural anesthesia by itself OR in combination with either sedation or general anesthesia. If spinal anesthesia is used to obtain neuraxial blockade, the spinal anesthetic will be induced prior to the insertion of the epidural catheter. If general anesthesia is used in combination with neuraxial blockade, the general anesthetic will be induced after the insertion of the epidural catheter. The attending anesthesiologist will determine the specific anesthetic and drugs.

Postoperative analgesia will be initiated in the recovery room. Each institution's postoperative epidural analgesia policy will dictate the specific drug formulation and rate but they will be very similar between sites. All five participating sites use mixtures of a long-acting local anesthetic (bupivacaine or ropivacaine) and narcotic (fentanyl, morphine, or hydromorphone) of varying concentrations (Table 7). *The goal will be to achieve and maintain a state of no or minimal postoperative pain in the patient with minimal side effects.* Because the beneficial effects of neuraxial blockade are mostly from local anesthetic drugs, the epidural analgesic formulation will always contain local anesthetic in this study. Adjunctive agents such as oral or rectal acetaminophen or nonsteroidal anti-inflammatory drugs may be added. Epidural analgesia will be maintained until the patient is able to tolerate oral intake AND pain relief is easily achieved using oral analgesics.

In some instances, attempts at insertion of an epidural catheter prior to surgery will fail. The number of attempts will be left to the discretion of the attending anesthesiologist. If the patient desires epidural analgesia, another attempt may be made at epidural catheter insertion as soon as it is feasible in the postoperative period. Similarly, in patients who received an epidural catheter, if the catheter is dislodged or is ineffective within the first 24 hours after surgery, the attending pain specialist may re-insert an epidural catheter. For patients in whom insertion is unsuccessful and for patients who decline re-insertion, analgesia will be continued with intravenous narcotic analgesia as in the control group (see below). Events in these patients will be counted against the epidural group on an intention-to-treat basis.

Control (general anesthesia and intravenous analgesia): Patients allocated to the control group will not receive an epidural catheter. General anesthesia will be administered intraoperatively with the specific drugs determined by the attending anesthesiologist. Postoperative analgesia will be obtained using intravenous narcotics. Each institution's postoperative narcotic analgesia policy will dictate the specific drug formulation, mode of administration, dose, and frequency, but these will be very similar between sites again (Table 7). Possible modes of administration include the patient-controlled analgesia (PCA) pump, nurse administered intermittent IV boluses, or continuous IV infusions. Adjunctive agents such as oral or rectal acetaminophen or

nonsteroidal anti-inflammatory drugs may be added. Adjustments to the IV analgesics will be at the discretion of the physician responsible for pain management. *Again, the goal will be to achieve and maintain a state of no or minimal postoperative pain in the patient with minimal side effects.* Intravenous narcotics will be continued until the patient is able to tolerate oral intake AND pain relief is easily achieved using oral analgesics.

Other management decisions
Aside from the interventions described above, intraoperative anesthetic management, monitoring, surgical technique, and other aspects of postoperative care will be left to the discretion of the attending physicians. The attending physician will be privy to any symptoms, bloodwork, and ECG results that are obtained by the POET investigators. All decisions regarding treatment of perioperative cardiorespiratory events, should they occur, will be at the discretion of the attending physician.

2.3. What are the proposed practical arrangements for allocating participants to trial groups?

One to two hours prior to surgery, the designated research coordinator (DRC) will log onto the secure study website of the coordinating central office (CCO), where randomisation will be performed using a computer-generated random number table with permuted random blocks stratified by hospital site. Randomisation will also be stratified by type of surgery (lower limb surgery, which would receive either lumbar epidural analgesia or IV narcotic analgesia AND non-lower limb surgery, which would receive either thoracic epidural analgesia or IV narcotic analgesia). The table and the block sizes will be unavailable to individuals involved in the recruitment or management of patients. Once patients are randomised, they will be followed and analysed within the group to which they are allocated (intention-to-treat analysis) regardless of whether they receive the assigned treatment or not.

2.4. What are the proposed methods for protecting against other sources of bias?

In theory, one could insert an epidural catheter into every research participant and use a placebo epidural infusion in those allocated to intravenous narcotics. Some believe this would be unethical and we have decided against this approach. As a result of this decision, the participants and their caregivers will not be blinded.

Investigators (physicians), who are not involved in the clinical care of the patients, will obtain data at the bedside (e.g. clinical signs from the respiratory examination). In order to blind the investigators, patients receiving IV narcotic analgesia will have an epidural catheter ("sham epidural") taped to the surface of their backs. Patients receiving epidural analgesia will already have an IV catheter, which is mandatory in clinical practice for patients receiving epidural analgesia. The tubings from the epidural and intravenous catheters and the analgesic delivery system will be covered so that the investigator will be unable to determine the patient's allocated analgesic. For hospital sites where delivery of epidural analgesics uses a delivery system that is different from the one for delivery of IV narcotic analgesics, the patients will have both delivery systems at the bedside (Figure 5a) but only one will be connected to the patient. During clinical assessments by the blinded investigator, both delivery systems and the tubings will be covered (Figure 5b). The blinded investigator will also interpret the daily ECGs and troponin values, which will be obtained from the patient's chart by a separate unblinded DRC.

As we plan to collect data on crossover rates and details on the anesthetics, analgesics, and surgical procedures, the unblinded DRC will collect data from the patient's medication record and chart. The unblinded DRC will enter all of the data, including the data collected by the

blinded investigator, to avoid unblinding the investigator performing the blinded patient assessments. All other personnel who are not involved in the care of the patient (outcome adjudicators, and data analysts) will be blinded.

2.5. What are the planned inclusion / exclusion criteria?

Table 8 summarises the eligibility criteria. Based on the few studies that examine cardiac and respiratory risk together in patients undergoing non-cardiopulmonary surgery, high cardiac risk, chronic obstructive pulmonary disease (COPD), and duration of anesthesia of at least three hours are predictors of increased cardiorespiratory risk.[38,130,131] We conducted a careful analysis of perioperative cardiac risk for the POISE Study. This resulted in inclusion criteria that maximise the number of eligible patients while maintaining a high control event rate for cardiac risk based on data courtesy of Drs. Lee Goldman[8] and Ken Gilbert[17]. Our eligibility criteria incorporate the inclusion criteria from the POISE Study (for high cardiac risk), COPD, and duration of anesthesia. Patients will be excluded if they are pregnant, have contraindications to the proposed anesthetic or analgesic interventions, have undergone coronary artery revascularisation with no evidence of subsequent cardiac ischemia, have respiratory complications at the time of surgery, or have an anticipated life expectancy of less than 30 days.

2.6. What is the proposed duration of treatment period?

Interim data from our POISE Study reveal that 67% of perioperative MIs occur in the first 48 hours after surgery; ideally, epidural or intravenous analgesia should be maintained for at least that period of time. In practice, not all surgical procedures will require such intense pain relief for that duration and the need to continue epidural or intravenous analgesia is evaluated on a day-to-day basis. Park *et al.* noted that epidural analgesia was maintained for an average of 55.2 hours in their RCT, which did not specify a minimum duration of treatment period.[124] For this pilot study, we will maintain epidural or intravenous analgesia for a *minimum* of 24 postoperative hours, although we expect that the duration will exceed this minimum period. By using a shorter time period in this pilot study, we will maximise the number of eligible patients and will be able to determine the types of procedures in which epidural or intravenous analgesia could be maintained feasibly for 24 hours, 48 hours, and 72 hours after surgery.

2.7. What is the proposed frequency and duration of follow-up?

We will assess patients daily after surgery until hospital discharge. They will be contacted by telephone, if already discharged, 30 days after surgery for a final follow-up interview. The total duration of follow-up will be 30 days.

2.8. What are the proposed primary and secondary outcome measures?

Primary outcome measures for the pilot study

Our primary outcome measures for the pilot study are the rates of enrolment, follow-up, and crossover at the participating centres. We will consider recruitment feasible for a large multicentre RCT if we are able to enrol 250 patients (50 patients per site) over 12 months. We will consider the pilot study successful if we are able to enrol =70% of all eligible patients, we can achieve complete follow-up in =95% (at least 238 / 250) of all enrolled patients, and =5% of all enrolled patients crossover from one modality to the other.

Secondary outcome measures for the pilot study (Table 9)
The secondary outcome measures for the pilot study will be the clinical outcome measures that will be used if a large multicentre RCT is feasible (see below). The detailed definitions for these outcomes are summarized in Table 9.

The primary outcome for a large multicentre RCT will be a combined 30-day outcome of all-cause mortality, nonfatal MI, cardiac arrest, postoperative pneumonia, and respiratory failure. Secondary outcomes will include DVT, pulmonary embolism, transient ischemic attack, stroke, and congestive heart failure during the first 30 postoperative days. Safety outcomes will be clinically significant bradycardia, and clinically significant hypotension during the period in which postoperative epidural or narcotic analgesia is used. Rare complications, such as epidural hematoma, epidural abscess, and neuropraxia will be sought prospectively also.

Adjudication of outcomes
All outcomes will be adjudicated by a blinded outcomes adjudication committee, consisting of anesthesiologists, internists, cardiologists, and respirologists. The committee will review the documentation, request any additional necessary information, and make the final judgment regarding each event. Disagreement will be resolved by consensus.

2.9. How will the outcome measures be measured at follow-up? (Appendix)
The case report forms to be used in this pilot study are enclosed in the Appendix.

Primary outcome measures for the pilot study
To determine recruitment rate, each site's DRC will maintain a screening log of all eligible patients, the eligible patients who were not recruited and the reasons for their lack of participation (e.g. refusal from patient, anesthesiologist, or surgeon), and the patients who were successfully recruited into the study. For all patients enrolled in the study, the DRC will record the success or failure in contacting the patients for their final interview 30 days after surgery.

Details on the anesthetic (drugs doses and times), the neuraxial blockade if applicable (level of epidural or spinal; drug concentrations, doses, and times), and the analgesic (drugs doses and times) will be collected immediately after surgery and then daily beginning on the first postoperative day while the patients are receiving epidural or IV narcotic analgesia.

To ensure that postoperative analgesia has been achieved and is comparable between the two groups, postoperative pain intensity, at rest and with movement, will be measured using a 0 to 10 visual analogue scale (VAS; 0 = no pain, 10 = worst possible pain) every four hours after surgery while the patients are receiving epidural or IV narcotic analgesia.

Secondary outcome measures for the pilot study
We will also record the outcomes that will be used for a large multicentre RCT. An electrocardiogram (ECG) will be recorded 6 to 12 hours postoperatively and on the first, second, and 30th day after surgery. Troponin I will be drawn 6 to 12 hours postoperatively and on the first, second, and third day after surgery. If MI is suspected, centres are encouraged to obtain more frequent ECGs and cardiac enzymes as clinically necessary. Chest examination (percussion and auscultation) and chest radiograph will be obtained if there is a clinical suspicion of pneumonia, suggested by a new cough, sputum, dyspnea, fever, altered mental status, or abnormalities of the white blood cell count or arterial blood gas. Additional tests and interventions will be at the discretion of the patient's physician. The DRC will record any co-interventions (e.g. nasogastric decompression, incentive spirometry, and chest physiotherapy) administered to the patient. The DRC will review the patients' charts for any outcomes prior to

hospital discharge. At 30 days after surgery, patients will be contacted by telephone for an interview and an ECG. If patients indicate they have experienced an outcome within the first 30 days after surgery, their physician will be contacted to acquire the appropriate documentation.

2.10. Will health services research issues be addressed?

This pilot study will not address any health services issues such as economics or quality of life. If a large multicentre trial is shown to be feasible, evaluation of economic outcomes and quality of life measures may be incorporated into the full-scale RCT.

2.11. What is the proposed sample size?

Sample size for the pilot study

For this pilot study, we have chosen five centres with established postoperative epidural and IV narcotic analgesia policies and high volumes of surgical procedures. As the recruitment rate may be influenced by operating room closures during certain times of the year (e.g. during spring break and the summer months and at the end of the year), we have chosen to carry out the study over a one-year period to obtain an accurate estimate of the recruitment rate. Thus, our proposed sample size for this pilot study is 250 patients (50 patients per site).

Sample size estimates for a large multicentre trial

Based on our pooled estimates of the rates of postoperative cardiac death, myocardial infarction, and pneumonia, we estimate that the control event rate will range from 15 to 25% for the primary outcome to be used in a large multicentre RCT. Sample size calculations are presented in Table 10 for control event rates in this range, clinically plausible relative risk reductions from 20 to 30%, type I error of 5%, and power from 80 to 90%.

A recent survey of Canadian academic departments of anesthesia regarding their use of neuraxial blockade and their willingness to participate in a multicentre perioperative epidural RCT showed that epidural use is widespread with the respondents utilising epidural techniques in over 21000 patients annually.[136] Departments indicated that over 50% of these patients would be available for recruitment in a clinical trial. Thirty centres have indicated an interest in participating in an epidural trial. Assuming each centre participates and is able to recruit 50 patients per year (~1 patient per week), 1500 patients could be recruited annually.

2.12. What is the planned recruitment rate?

Our goal is to recruit one patient per site per week (5 patients per week). Recruitment rates in recent epidural trials have varied from 0.5 to 3.5 patients per site per month (Table 5); therefore, one of the goals of this pilot study is to obtain an accurate estimate of the recruitment rate.

We will recruit patients in preoperative assessment clinics. All patients will be screened by the DRC to determine their eligibility. In addition, medical staff identifying a potential patient will page the site's DRC, who will confirm patient eligibility and provide the patient with the subject information and consent form for consideration. All eligible patients will be approached. Informed consent will be sought two to four hours prior to surgery.

2.13. Are there likely to be any problems with compliance?

We do not anticipate a problem with compliance as our proposed treatment and control interventions are similar to current clinical practices; however, we do expect that some patients will cross over from epidural analgesia to IV narcotic analgesia and *vice versa*. Our goal is to attain a crossover rate of =5%. Determination of the crossover rate is one of the outcomes of this pilot study.

Ideally, a crossover rate =2% would be preferable; however, in the two largest RCTs comparing epidural and IV narcotic analgesia, which were the only studies to report crossover rates, 6.5% of participants switched from epidural analgesia to IV narcotic analgesia[124,125] and 4.3%[125] to 9.7%[124] of participants switched from IV narcotic analgesia to epidural analgesia. Overall crossover rates were 8.1%[124] and 5.4%[125] respectively (Table 5). In clinical practice, inadequate or failed epidural analgesia ranges from 5 to 10%. Thus, we believe our goal is a realistic one for a crossover rate in an epidural trial.

2.14. What is the likely rate of loss to follow-up?

Given the 30-day follow-up, we do not anticipate more than 5% loss to follow-up. Determination of the rate of loss to follow-up is one of the outcomes of this pilot study.

2.15. How many centres will be involved?

We will conduct the pilot study at five acute-care hospitals: the Hamilton Health Sciences, the Ottawa Hospital, Toronto General Hospital, Toronto Western Hospital, and Vancouver General Hospital. All five sites have pre-existing policies for the initiation and maintenance of postoperative epidural and intravenous narcotic analgesia, which are managed by anesthesiologists within the acute pain service.

2.16. Details of the planned analyses

Data management

The Centre for Clinical Epidemiology and Evaluation (C2E2) at the Vancouver Coastal Health Research Institute will be the CCO and will provide methodological support for this pilot study. All demographic and personal data on the research participants will be treated as confidential information and will be stored on a high-security computer system with a firewall. Data will be recorded daily during each patient's hospital stay on electronic case report forms (CRFs), which will be completed by each site's DRC and stored in a SQL Server database at the CCO. Source documents will be stored locally in locked premises at each site. The cleaned data will be imported to SAS for analysis. All computer systems used in the study will be located in locked, high-security areas at each participating site.

Analysis of primary outcomes for the pilot study

Analysis will be performed in a blinded fashion. We will tabulate the types of operations that were amenable to postoperative epidural analgesia in which anesthesiologists, surgeons, and patients were willing to participate in this study. The recruitment rate will be calculated as the proportion of eligible patients, undergoing operations in which anesthesiologists and surgeons proved willing to enrol patients, and who provided informed consent. The frequency of crossover will be calculated for epidural-to-intravenous crossovers and intravenous-to-epidural crossovers regardless of the reason (e.g. failure of analgesic modality, patient request, etc).

Analysis of clinical outcomes (secondary outcomes) for the pilot study

As our objectives for this pilot study examine the feasibility of a larger clinical trial, it will be underpowered to evaluate the effect of neuraxial blockade on clinical outcomes; however, we will tabulate the number of clinical outcomes (primary, secondary, and safety outcomes) by treatment group. For discrete outcomes, we will compare rates of occurrence between the two groups using the log-rank statistic.

One interim analysis (see sections 2.17 and 3.3) of safety outcomes and a final analysis of all outcomes will be performed. For the interim analysis, the modified Haybittle-Peto rule of four

standard deviations will be used (for a corresponding $\alpha = 0.0001$). Should an intervention surpass this rule, then the independent, external data safety monitoring committee (DSMC) will recommend stopping the study. For the final analysis, we will use the conventional $\alpha = 0.05$.

2.17. What is the proposed frequency of analyses?
The independent, external DSMC will conduct one interim analysis of safety outcomes after 50% of the 30-day follow-up data are available (See section 3.3). We will analyse all of the data at the end of the study.

2.18. Are there any planned subgroup analyses?
We do not plan to perform subgroup analyses nor draw clinical conclusions based on this pilot study.

2.19. Has any pilot study been carried out using this design?
This proposal is a pilot study to determine the feasibility of answering our primary research question using a large multicentre RCT.

3. Trial Management

3.1. What are the arrangements for day to day management of the trial?
At each site, the DRC will be responsible for the recruitment of participants, the daily collection of unblinded data, and the completion of CRFs. The site investigator will be responsible for the daily collection of blinded data and the local administration of the trial. Questions relating to the study will be fielded by the DRC and the site investigator. Questions relating to the clinical care of the patient, including clinical questions regarding the postoperative analgesia, will be managed by the patient's primary physician and the acute pain service. The CCO will be responsible for site initiations, data checking, data cleaning, and database management. The co-principal investigators and site investigators will hold monthly teleconferences, coordinated by the CCO, to address questions that may arise during the conduct of this pilot study.

3.2. What will be the role of each principal applicant and co-applicant?
Dr. Peter Choi is the co-principal investigator and proposed principal applicant and will oversee the conduct of this pilot study. He is primarily responsible for the development of the study protocol and coordination of the study centres. He will be responsible, along with the staff at the CCO, in ensuring timely conduct of data internal consistency checks and data analysis, provision of data and supporting information to the adjudication committee, and liason with the steering committee, the DSMC, and Health Canada.

Dr. Scott Beattie is the other co-principal investigator. He and Drs. Vincent Chan, James Paul, Hamed Umedaly, and Homer Yang will be the site investigators at the Toronto General Hospital, Toronto Western Hospital, Hamilton Health Sciences, Vancouver General Hospital, and Ottawa Hospital respectively. They will be responsible for obtaining ethics approval from their local institutional research ethics boards (REBs); ensuring all physicians and nurses involved in the perioperative care of the patients are informed about this study via educational inservices, posters, and pocket protocols; confirming that all surgical patients are being screened, all eligible patients are invited to participate in this study, and all consented patients are randomised and followed appropriately; and ensuring that all CRFs are completed in an appropriate and timely fashion.

Dr. Keith Chambers is responsible for developing the randomisation scheme and will assist Dr. Choi in ensuring that the data internal consistency checks and data analysis are carried out

in a timely fashion. Drs. Norman Buckley (anesthesia), P.J. Devereaux (cardiology), Ashraf Fayad (anesthesia), and Mark Fitzgerald (respirology) will comprise the adjudication committee. Dr. York Hsiang will provide expertise relating to surgical questions that may arise during this study.

We believe that we have the experience to successfully complete the POET pilot study. Drs. Scott Beattie, Vincent Chan, Peter Choi, and Homer Yang have completed multicentre RCTs. Drs. Keith Chambers, Peter Choi, PJ Devereaux, Mark FitzGerald, and James Paul have training in clinical epidemiology and clinical trials methodology.

3.3. Trial steering committee and data safety and monitoring committee

The trial steering committee will consist of the principal applicants and co-applicants. An independent, external DSMC will ensure patient safety, prepare an interim analysis, ensure that the study is conducted at the highest ethical standards, and provide feedback to the steering committee. The DSMC will conduct one planned interim analysis after 50% of the 30-day follow-up data are available. If safety concerns arise at any time during the study, the DSMC chairperson will convene a formal meeting of the full committee and make recommendations after considering all available data from the study and other relevant studies.

4. Other Information

4.1. International collaboration

Our investigators can also draw on the collective expertise of the perioperative research community. Our research group consists of members of the Canadian Perioperative Research Network, which includes anesthesiologists, internists, and surgeons across Canada. Our group has obtained CIHR funding for the POISE Study. Dr. Choi is a member of the executive committee for the POISE Study; Drs. Yang and Devereaux are the co-principal investigators. We have also expanded our research group outside Canada and have investigators in 11 other countries.

4.2. Concurrent conduct of the POISE Study and the POET Pilot Study is more efficient

Our group's collaborative experience from conducting the POISE Study will help us in this pilot study. The timing is also appropriate because the selection criteria for both studies are similar and the areas where patients are screened will be identical; thus, we will be more efficient in our use of research staff and resources. Patients will participate in either the POISE Study or the POET Pilot Study. A large number of patients, who are at moderate- to high-risk for cardiac events, are already prescribed beta-blockers. These patients will *not* meet the criteria for the POISE Study but will be eligible for this pilot study. Patients who are eligible for both studies will be approached to enter the POISE Study first. Thus, the two studies will not compete with each other. Recruitment will be more efficient.

4.3. Ethics approval and reporting of serious adverse events

Currently, two sites (Hamilton Health Sciences and Vancouver General Hospital), have received ethics approval (Appendix). The study protocol and consent form are undergoing review from the local institutional REBs at the remaining sites. We will conduct the study in accordance to the ICH Guideline for Good Clinical Practice.[137] Unexpected or serious adverse events (SAEs) as defined by Health Canada will be reported in accordance to the Canadian Adverse Drug Reaction Monitoring Program guidelines. The CCO will be responsible for informing Health Canada and any other regulatory bodies. Adverse events and adjustments to the study protocol, if any, will be communicated to the REBs.

Bibliography

1. Mangano DT. Peri-operative cardiovascular morbidity: new developments. Ballieres Clin Anaesthesiol 1999; 13:335-48.
2. Arozullah AM, Shukri FK, Henderson WG, et al, for the Participants in the National Veterans Affairs Surgical Quality Improvement Program. Development and validation of a multifactorial risk index for predicting postoperative pneumonia after major noncardiac surgery. Ann Intern Med 2001; 135:847-57.
3. Kazmers A, Jacobs L, Perkins A. The impact of complications after vascular surgery in Veterans Affairs medical centers. J Surg Res 1997; 67:62-6.
4. Lawrence VA, Hilsenbeck SG, Mulrow CD, Dhanda R, Sapp J, Page CP. Incidence and hospital stay for cardiac and pulmonary complications after abdominal surgery. J Gen Intern Med 1995; 10:671-8.
5. Kappstein I, Schulgen G, Richtmann R, et al. [Prolongation of hospital stay by nosocomial pneumonia and wound infection]. Dtsch Med Wochenschr 1991; 116:281-7.
6. Mangano DT. Perioperative cardiac morbidity. Anesthesiology 1990; 72:153-84.
7. Wallace WC, Cinat ME, Nastanski F, Gornick WB, Wilson SE. New epidemiology for postoperative nosocomial infections. Am Surg 2000; 66:874-8.
8. Goldman L, Caldera DL, Nussbaum SR, et al. Multifactorial index of cardiac risk in noncardiac surgical procedures. N Engl J Med 1977; 297:845-50.
9. Zeldin RA, Assessing cardiac risk in patients who undergo noncardiac surgical procedures. Can J Surg 1984; 27:404-4.
10. Larsen SF, Olsen KH, Jacobsen E, et al. Prediction of cardiac risk in noncardiac surgery. Eur Heart J 1987; 8:179-85.
11. Pedersen T. Complications and death following anaesthesia. A prospective study with special reference to the influence of patient-, anaesthesia-, and surgery-related risk factors. Dan Med Bull 1994; 41:319-31.
12. Lee TH, Marcantonio ER, Mangione CM, et al. Derivation and prospective validation of a single index for cardiac risk of major noncardiac surgery. Circulation 1999; 100:1043-9.
13. Detsky AS, Abrams HB, McLaughlin JR, et al. Predicting cardiac complications in patients undergoing non-cardiac surgery. J Gen Intern Med 1986; 1:211-9.
14. Mangano DT, Browner WS, Hollenberg M, et al. Association of perioperative myocardial ischemia with cardiac morbidity and mortality in men undergoing noncardiac surgery. N Engl J Med 1990; 323:1781-8.
15. Shah KB, Kleinman BS, Rao TL, Jacobs HK, Mestan K, Schaafsma M. Angina and other risk factors in patients with cardiac diseases undergoing noncardiac operations. Anesth Analg 1990; 70:240-7.
16. Ashton CM, Petersen NJ, Wray NP, et al. The incidence of perioperative myocardial infarction in men undergoing noncardiac surgery. Ann Intern Med 1993; 118:504-10.
17. Gilbert K, Larocque BJ, Patrick LT. Prospective evaluation of cardiac risk indices for patients undergoing noncardiac surgery. Ann Intern Med 2000; 133:356-9.
18. Lette J, Waters D, Cerino M, Picard M, Champagne P, Lapointe J. Preoperative coronary artery disease risk stratification based on dipyridamole imaging and a simple three-step, three-segment model for patients undergoing noncardiac vascular surgery or major general surgery. Am J Cardiol 1992; 69:1553-8.
19. Brown KA, Rowen M. Extent of jeopardized viable myocardium determined by myocardial perfusion imaging best predicts perioperative cardiac events in patients undergoing noncardiac surgery. J Am Coll Cardiol 1993; 21:325-30.

20. Halm EA, Browner WS, Tubau JF, Tateo IM, Mangano DT. Echocardiography for assessing cardiac risk in patients having noncardiac surgery. Ann Intern Med 1996; 125:433-41.
21. McPhail N, Menkis A, Shariatmadar A, et al. Statistical prediction of cardiac risk in patients who undergo vascular surgery. Can J Surg 1985; 28:404-6.
22. Raby KE, Goldman L, Creager MA, et al. Correlation between preoperative ischemia and major cardiac events after peripheral vascular surgery. N Engl J Med 1989; 321:1296-1300.
23. Pasternack PF, Grossi EA, Baumann FG, et al. Silent myocardial ischemia monitoring predicts late as well as perioperative cardiac events in patients undergoing vascular surgery. J Vasc Surg 1992; 16:171-80.
24. Raby KE, Barry J, Creager MA, Cook EF, Weisberg MC, Goldman L. Detection and significance of intraoperative and postoperative myocardial ischemia in peripheral vascular surgery. JAMA 1992; 268:222-7.
25. Jivegard L, Frid I, Haljamae H, Hallenberg B, Holm J, Johansson CR. Cardiac risk and peripheral vascular surgery: new approach based on a multifactorial risk index. Br J Surg 1993; 80:354-8.
26. Landesberg G, Luria MH, Cotev S, et al. Importance of long-duration postoperative ST-segment depression in cardiac morbidity after vascular surgery. Lancet 1993; 341:715-9.
27. Hendel RC, Whitfield SS, Villegas BJ, Cutler BS, Leppo JA. Prediction of late cardiac events by dipyridamole thallium imaging in patients undergoing elective vascular surgery. Am J Cardiol 1992; 70:1243-9.
28. Davila-Roman VG, Waggoner AD, Sicard GA, Geltman EM, Schechtman KB, Perez JE. Dobutamine stress echocardiography predicts surgical outcome in patients with an aortic aneurysm and peripheral vascular disease. J Am Coll Cardiol 1993; 21:957-63.
29. McFalls EO, Doliszny KM, Grund F, Chute E, Chesler E. Angina and persistent exercise thallium defects: independent risk factors in elective vascular surgery. J Am Coll Cardiol 1993; 21:1347-52.
30. Poldermans D, Fioretti PM, Forster T, et al. Dobutamine stress echocardiography for assessment of perioperative cardiac risk in patients undergoing major vascular surgery. Circulation 1993; 87:1506-12.
31. Baron JF, Mundler O, Bertrand M, et al. Dipyridamole-thallium scintigraphy and gated radionuclide angiography to assess cardiac risk before abdominal aortic surgery. N Engl J Med 1994; 330:663-9.
32. Bry JD, Belkin M, O'Donnell TF, et al. An assessment of the positive predictive value and cost-effectiveness of dipyridamole myocardial scintigraphy in patients undergoing vascular surgery. J Vasc Surg 1994; 19:112-24.
33. L'Italien GJ, Paul SD, Hendel RC, et al. Development and validation of a Bayesian model for perioperative cardiac risk assessment in a cohort of 1,801 vascular surgical candidates. J Am Coll Cardiol 1996; 27:779-86.
34. Wightman JAK. A prospective survey of the incidence of postoperative pulmonary complications. Br J Surg 1968; 55:85-91.
35. Ti TK, Yong NK. Postoperative pulmonary complications – a prospective study in the tropics. Br J Surg 1974; 61:49-52.
36. Appleberg M, Gordon L, Fatti LP. Preoperative pulmonary evaluation of surgical patients using the vitalograph. Br J Surg 1974; 61:57-9.
37. Järhult J, Sandhammar B. Postoperative infections in the small hospital. A prospective one-year study. Acta Chir Scand 1981; 147:325-9.
38. Pedersen T, Eliasen K, Henriksen E. A prospective study of risk factors and cardiopulmonary complications associated with anaesthesia and surgery: risk indicators of cardiopulmonary morbidity. Acta Anaesthesiol Scand 1990; 34:144-55.

39. Wong DH, Weber EC, Schell MJ, Wong AB, Anderson CT, Barker SJ. Factors associated with postoperative pulmonary complications in patients with severe chronic obstructive pulmonary disease. Anesth Analg 1995; 80:276-84.
40. Mitchell CK, Smoger SH, Pfeifer MP, et al. Multivariate analysis of factors associated with postoperative pulmonary complications following general elective surgery. Arch Surg 1998; 133:194-8.
41. de Albuquerque Medeiros R, Faresin SM, Jardim JR. [Postoperative lung complications and mortality in patients with mild-to-moderate COPD undergoing elective general surgery]. Arch Bronconeumol 2001; 37:227-34.
42. Daschner FD, Frey P, Wolff G, Baumann PC, Suter P. Nosocomial infections in intensive care wards: a multicenter prospective study. Intensive Care Med 1982; 8:5-9.
43. Donowitz LG, Wenzel RP, Hoyt JW. High risk of hospital-acquired infection in the ICU patient. Crit Care Med 1982; 10:355-7.
44. Konrad F, Wiedeck H, Kilian J, Deller A. [Risk factors for nosocomial pneumonia in intensive care patients. A prospective study to identify "high-risk" patients]. Anaesthesist 1991; 40:483-90.
45. Latimer RG, Dickman M, Day WC, Gunn ML, DuWayne Schmidt C. Ventilatory patterns and pulmonary complications after upper abdominal surgery determined by preoperative and postoperative computerized spirometry and blood gas analysis. Am J Surg 1971; 122:622-32.
46. Grundmann R, Weber F, Pichlmaier H. [Surgical quality control by a simple complication score]. Langenbecks Arch Chir 1987; 371:3-12.
47. Lumbreras C, Lizasoain M, Moreno E, et al. Major bacterial infections following liver transplantation: a prospective study. Hepatogastroenterology 1992; 39:362-5.
48. Williams-Russo P, Charlson ME, MacKenzie CR, Gold JP, Shires GT. Predicting postoperative pulmonary complications. Is it a real problem? Arch Intern Med 1992; 152:1209-13.
49. Ephgrave KS, Kleiman-Wexler R, Pfaller M, Booth B, Werkmeister L, Young S. Postoperative pneumonia: A prospective study of risk factors and morbidity. Surgery 1993; 114:815-21.
50. Liedman BL, Bennegård K, Olbe LC, Lundell LR. Predictors of postoperative morbidity and mortality after surgery for gastro-oesophageal carcinomas. Eur J Surg 1995; 161:173-80.
51. Hall JC, Tarala RA, Hall JL. Respiratory insufficiency after abdominal surgery. Respirology 1996; 1:133-8.
52. Brooks-Brunn JA. Predictors of postoperative pulmonary complications following abdominal surgery. Chest 1997; 111:564-71.
53. Delgado-Rodríguez M, Medina-Cuadros M, Martínez-Gallego G, Sillero-Arenas M. Usefulness of intrinsic surgical wound infection risk indices as predictors of postoperative pneumonia risk. J Hosp Infect 1997; 35:269-76.
54. Mathew JT, D'Souza GA, Kilpadi AB. Respiratory complications in postoperative patients. J Assoc Physicians India 1999; 47:1086-8.
55. Whooley BP, Law S, Murthy SC, Alexandrou A, Wong J. Analysis of reduced death and complication rates after esophageal resection. Ann Surg 2001; 233:338-44.
56. Johnston KW. Multicenter prospective study of nonruptured abdominal aortic aneurysm. Part II. Variables predicting morbidity and mortality. J Vasc Surg 1989; 9:437-47.
57. Svensson LG, Hess KR, Coselli JS, Safi HJ, Crawford ES. A prospective study of respiratory failure after high-risk surgery on the thoracoabdominal aorta. J Vasc Surg 1991; 14:271-82.
58. Cucchiara RF, Benefiel DJ, Matteo RS, DeWood M, Albin MS. Evaluation of esmolol in controlling increases in heart rate and blood pressure during endotracheal intubation in patients undergoing carotid endarterectomy. Anesthesiology 1986; 65:528-31.
59. Magnusson J, Thulin T, Werner O, Järhult J, Thomson D. Hemodynamic effects of pretreatment with metoprolol in hypertensive patients undergoing surgery. Br J Anaesth 1986; 58:251-60.

60. Stone G, Foëx P, Sear JW, Johnson LL, Khambatta H, Triner L. Myocardial ischemia in untreated hypertensive patients: effect of a single small oral dose of a beta-adrenergic blocking agent. Anesthesiology 1988; 68:495-500.
61. Mangano DT, Layug EL, Wallace A, et al, for the Multicenter Study of Perioperative Ischemia Research Group. Effect of atenolol on mortality and cardiovascular morbidity after noncardiac surgery. N Engl J Med 1996; 335:1713-20.
62. Jakobsen C-J, Bille S, Ahlburg P, Rybro L, Hjortholm K, Andressen EB. Perioperative metoprolol reduces the frequency of atrial fibrillation after thoracotomy for lung resection. J Cardiothorac Vasc Anesth 1997; 11:746-51.
63. Wallace A, Layug B, Tateo I, et al, for the McSPI Research Group. Prophylactic atenolol reduces postoperative myocardial ischemia. Anesthesiology 1998; 88:7-17.
64. Bayliff CD, Massel DR, Inculet RI, et al. Propranolol for the prevention of postoperative arrhythmias in general thoracic surgery. Ann Thorac Surg 1999; 67:182-6.
65. Poldermans D, Boersma E, Bax JJ, et al. The effect of bisoprolol on perioperative mortality and myocardial infarction in high-risk patients undergoing vascular surgery. N Engl J Med 1999; 341:1789-94.
66. Raby KE, Brull SJ, Timimi F, et al. The effect of heart rate control on myocardial ischemia among high-risk patients after vascular surgery. Anesth Analg 1999; 88:477-82.
67. Zaugg M, Tagliente T, Lucchinetti E, et al. Beneficial effects from ß-adrenergic blockade in elderly patients undergoing noncardiac surgery. Anesthesiology 1999; 91:1674-86.
68. Urban MK, Markowitz SM, Gordon MA, Urquhart BL, Kligfield P. Postoperative prophylactic administration of β-adrenergic blockers in patients at risk for myocardial ischemia. Anesth Analg 2000; 90:1257-61.
69. Stevens RD, Burri H, Tramèr MR. Pharmacologic myocardial protection in patients undergoing noncardiac surgery: a quantitative systematic review. Anesth Analg 2003; 97:623-33.
70. du Toit HJ, Weich HF, Weymar HW, Przybojewski JZ. Effects of nifedipine on the peri-operative ECG, as determined by continuous Holter monitoring. A double-blind study. S Afr Med J 1986; 69:427-31.
71. Retamal O, Coriat P, Pamela F, Godet G, Bertrand M, Viars P. [Prevention of hypertensive attacks after carotid surgery. The value of nifedipine and diltiazem]. Ann Fr Anesth Reanim 1986; 5:278-86.
72. Godet G, Coriat P, Baron JF, et al. Prevention of intraoperative myocardial ischemia during noncardiac surgery with intravenous diltiazem: a randomized trial versus placebo. Anesthesiology 1987; 66:241-5.
73. Caramella JP, Goursot G, Carcone B, et al. [Prevention of per- and postoperative myocardial ischemia in non-cardiac surgery by intravenous diltiazem]. Ann Fr Anesth Reanim 1988; 7:245-50.
74. Goldberg ME, Halpern N, Krakoff L, et al. Efficacy and safety of intravenous nicardipine in the control of postoperative hypertension. Chest 1991; 99:393-8.
75. Lindgren L, Lepantalo M, von Knorring J, Rosenberg P, Orko R, Schenin B. Effect of verapamil on right ventricular pressure and atrial tachyarrhythmia after thoracotomy. Br J Anaesth 1991; 66:205-11.
76. Amar D, Roistacher N, Burt ME, et al. Effects of diltiazem versus digoxin on dysrhythmias and cardiac function after pneumonectomy. Ann Thorac Surg 1997; 63:1374-81.
77. Hatziantoniou GP, Tassiopoulos PA, Fakiolas CN, et al. Perioperative risk of coronary patients undergoing major urological operations. Hellen J Cardiol 1997; 38:28-34.
78. Garcia-Guasch R, Llubia C, Preciado MJ, Costa A, Sust M, Vidal F. [Diltiazem compared with placebo in the prevention of myocardial ischemia during non-cardiac surgery]. Rev Esp Anest Reanim 1998; 45:409-15.

79. Amar D, Roistacher N, Rusch VW, et al. Effects of diltiazem prophylaxis on the incidence and clinical outcome of atrial arrhythmias after thoracic surgery. J Thorac Cardiovasc Surg 2000; 120:790-8.
80. Dorman T, Thompson DA, Breslow MJ, Lipsett PA, Rosenfeld BA. Nicardipine versus nitroprusside for breakthrough hypertension following carotid endarterectomy. J Clin Anesth 2001; 13:16-9.
81. Wijeysundera DN, Beattie WS. Calcium channel blockers for reducing cardiac morbidity after noncardiac surgery: a meta-analysis. Anesth Analg 2003; 97:634-41.
82. Coriat P, Daloz M, Bousseau D, Fusciari J, Echter E, Viars P. Prevention of intraoperative myocardial ischemia during noncardiac surgery with intravenous nitroglycerin. Anesthesiology 1984; 61:193-6.
83. Fusciardi J, Godet G, Bernard JM, Bertrand M, Kieffer E, Viars P. Roles of fentanyl and nitroglycerin in prevention of myocardial ischemia associated with laryngoscopy and tracheal intubation in patients undergoing operations of short duration. Anesth Analg 1986; 65:617-24.
84. Dodds TM, Stone JG, Coromilas J, Weinberger M, Levy DG. Prophylactic nitroglycerin infusion during noncardiac surgery does not reduce perioperative ischemia. Anesth Analg 1993; 76:705-13.
85. Divatia JV, Badwe RA, Juvekar R. Prophylactic nitroglycerin and perioperative ischemia: power analysis and a meta-analysis. Anesth Analg 1994; 79:1017-27.
86. Taylor DW, Barnett HJ, Haynes RB, et al. Low-dose and high-dose acetylsalicyclic acid for patients undergoing carotid endarterectomy: a randomised controlled trial. ASA and Carotid Endarterectomy (ACE) Trial Collaborators. Lancet 1999; 353:2179-84.
87. Efficacy of oral anticoagulants compared with aspirin after infrainguinal bypass surgery (The Dutch Bypass Oral Anticoagulants or Aspirin Study): a randomised trial. Lancet 2000; 355:346-51.
88. Pulmonary Embolism Prevention (PEP) Trial Collaborative Group. Prevention of pulmonary embolism and deep vein thrombosis with low dose aspirin: Pulmonary Embolism Prevention (PEP) Trial. Lancet 2000; 355:1295-302.
89. Antiplatelet Trialists' Collaboration. Collaborative overview of randomised trials of antiplatelet therapy – II: Maintenance of vascular graft or arterial patency by antiplatelet therapy. BMJ 1994; 308:159-68.
90. Tangelder MJD, Lawson JA, Algra A, Eikelboom BC. Systematic review of randomized controlled trials of aspirin and oral anticoagulants in the prevention of graft occlusion and ischemic events after infrainguinal bypass surgery. J Vasc Surg 1999; 30:701-9.
91. Girolami B, Bernardi E, Prins MH, et al. Antiplatelet therapy and other interventions after revascularization procedures in patients with peripheral arterial disease: a meta-analysis. Eur J Vasc Endovasc Surg 2000; 19:370-80.
92. Robless P, Mikhailidis DP, Stansby G. Systematic review of antiplatelet therapy for the prevention of myocardial infarction, stroke or vascular death in patients with peripheral vascular disease. Br J Surg 2001; 88:787-800.
93. Ellis JE, Drijvers G, Pedlow S, et al. Premedication with oral and transdermal clonidine provides safe and efficacious postoperative sympatholysis. Anesth Analg 1994; 79:1133-40.
94. Talke P, Li J, Jain U, et al, and the Study of Perioperative Ischemia Research Group. Effects of perioperative dexmedetomidine infusion in patients undergoing vascular surgery. Anesthesiology 1995; 82:620-33.
95. Stühmeier K-D, Mainzer B, Cierpka J, Sandmann W, Tarnow J. Small, oral dose of clonidine reduces the incidence of intraoperative myocardial ischemia in patients having vascular surgery. Anesthesiology 1996; 85:706-12.
96. McSPI-Europe Research Group. Perioperative sympatholysis. Beneficial effects of the alpha 2-adrenoceptor agonist mivazerol on hemodynamic stability and myocardial ischemia. Anesthesiology 1997; 86:346-63.

97. Oliver MF, Goldman L, Julian DG, Holme I, for the Mivazerol Trial Investigators Research Group. Effect of mivazerol on perioperative cardiac complications during non-cardiac surgery in patients with coronary heart disease. The European Mivazerol Trial (EMIT). Anesthesiology 1999; 91:951-61.
98. Matot I, Sichel JY, Yofe V, Gozal Y. The effect of clonidine premedication on hemodynamic responses to microlaryngoscopy and rigid bronchoscopy. Anesth Analg 2000; 91:828-33.
99. Nishina K, Mikawa K, Uesugi T, et al. Efficacy of clonidine for prevention of perioperative myocardial ischemia. A critical appraisal and meta-analysis of the literature. Anesthesiology 2002; 96:323-9.
100. Wijeysundera DN, Naik JS, Beattie WS. Alpha-2 adrenergic agonists to prevent perioperative cardiovascular complications: a meta-analysis. Am J Med 2003; 114:742-52.
101. Wallace AW, Galindez D, Salahieh A, et al. Effect of clonidine on cardiovascular morbidity and mortality after noncardiac surgery. Anesthesiology 2004; 101:284-93.
102. Møller A, Villebro N, Pedersen T, Tønnesen H. Effect of preoperative smoking intervention on postoperative complications: a randomised clinical trial. Lancet 2002; 359:114-7.
103. Thomas JA, McIntosh JM. Are incentive spirometry, intermittent positive pressure breathing, and deep breathing exercises effective in the prevention of postoperative pulmonary complications after upper abdominal surgery? A systemic overview and meta-analysis. Phys Ther 1994; 74:3-10.
104. Cheatham ML, Chapman WC, Key SP, Sawyers JL. A meta-analysis of selective *versus* routine nasogastric decompression after elective laparotomy. Ann Surg 1995; 221:469-78.
105. Kollef MH. The role of selective digestive tract decontamination on mortality and respiratory tract infections. A meta-analysis. Chest 1994; 105:1101-8.
106. D'Amico R, Pifferi S, Leonetti C, Torri V, Tinazzi A, Liberati A, on behalf of the study investigators. Effectiveness of antibiotic prophylaxis in critically ill adult patients: systematic review of randomised controlled trials. BMJ 1998; 316:1275-85.
107. Nathens AB, Marshal JC. Selective decontamination of the digestive tract in surgical patients. A systematic review of the evidence. Arch Surg 1999; 134:170-6.
108. Heys SD, Walker LG, Smith I, Eremin O. Enteral nutritional supplementation with key nutrients in patients with critical illness and cancer. A meta-analysis of randomized controlled clinical trials. Ann Surg 1999; 229:467-77.
109. Cook DJ, Reeve BK, Guyatt GH, et al. Stress ulcer prophylaxis in critically ill patients. Resolving discordant meta-analyses. JAMA 1996; 275:308-14.
110. Yang H, Raymer K, Butler R, Parlow J, Roberts R. Metoprolol after vascular surgery (MaVS). Can J Anesth 2004; 51:A7.
111. Brown DL. Spinal, epidural, and caudal anesthesia. In: Miller RD, ed. Anesthesia, 5th edition. Philadelphia: Churchill Livingstone; 2000: 1493.
112. Halter JB, Pflug AE. Relationship of impaired insulin secretion during surgical stress to anesthesia and cathecholamine release. J Clin Endocrinol Metab 1980; 51:1093-8.
113. Breslow MJ, Jordan DA, Christopherson R, et al. Epidural morphine decreases postoperative hypertension by attenuating sympathetic nervous system hyperactivity. JAMA 1989; 261:3577-81.
114. Blomberg S, Emanuelsson H, Kvist H, et al. Effects of thoracic epidural anesthesia on coronary arteries and arterioles in patients with coronary artery disease. Anesthesiology 1990; 73:840-7.
115. Liu S, Carpenter RL, Neal JM. Epidural anesthesia and analgesia. Their role in postoperative outcome. Anesthesiology 1995; 82:1474-506.
116. Rosenfeld BA, Beattie C, Christopherson R, et al, and the Perioperative Ischemia Randomized Anesthesia Trial Study Group. The effects of different anesthetic regimens on fibrinolysis and the development of postoperative arterial thrombosis. Anesthesiology 1993; 79:435-443.

117. Modig J, Borg T, Bagge L, Saldeen T. Role of extradural and of general anaesthesia in fibrinolysis and coagulation after total hip replacement. Br J Anaesth 1983; 55:625-9.
118. Davis FM, McDermott E, Hickton C, et al. Influence of spinal and general anaesthesia on haemostasis during total hip arthroplasty. Br J Anaesth 1987; 59:561-571.
119. Donadoni R, Baele G, Devulder J, Rolly G. Coagulation and fibrinolytic parameters in patients undergoing total hip replacement: influence of the anaesthesia technique. Acta Anaesthesiol Scand 1989; 33:588-592.
120. McCarthy GS. The effects of thoracic extradural analgesia on pulmonary gas distribution, functional residual capacity, and airway closure. Br J Anaesth 1976; 48:243-7.
121. Weller R, Rosenblum M, Conrad P, Gross JA. Comparison of epidural and patient controlled intravenous morphine following joint replacement surgery. Can J Anaesth 1991; 38:582-6.
122. Mankikian B, Cantineau JP, Bertrand M, Kieffer E, Sartene R, Viars P. Improvement of diaphragmatic function by a thoracic extradural block after upper abdominal surgery. Anesthesiology 1988; 68:379-86.
123. Sydow FW. The influence of anesthesia and postoperative analgesic management on lung function. Acta Chir Scand 1988; 55(Suppl):159-68.
124. Park WY, Thompson JS, Lee KK, and the Department of Veterans Affairs Cooperative Study #345 Study Group. Effect of epidural anesthesia and analgesia on perioperative outcomes. A randomized, controlled Veterans Affairs Cooperative Study. Ann Surg 2001; 234:560-71.
125. Rigg JRA, Jamrozik K, Myles PS, et al, for the MASTER Anaesthesia Trial Study Group. Epidural anaesthesia and analgesia and outcome of major surgery: a randomised trial. Lancet 2002; 359:1276-82.
126. Rodgers A, Walker N, Schug S, et al. Reduction of postoperative mortality and morbidity with epidural or spinal anaesthesia: results from overview of randomised trials. BMJ 2000; 321:1-12.
127. Beattie WS, Badner NH, Choi P. Epidural analgesia reduces postoperative myocardial infarction: a meta-analysis. Anesth Analg 2001; 93:853-8.
128. Beattie WS, Badner NH, Choi PT. Meta-analysis demonstrates statistically significant reduction in postoperative myocardial infarction with the use of thoracic epidural analgesia. Anesth Analg 2003; 97:919-20.
129. Ballantyne JC, Carr DB, deFerranti S, et al. The comparative effects of postoperative analgesic therapies on pulmonary outcome: cumulative meta-analyses of randomized, controlled trials. Anesth Analg 1998; 86:598-612.
130. Gerson MC, Hurst JM, Hertzberg VS, et al. Prediction of cardiac and pulmonary complications related to elective abdominal and noncardiac thoracic surgery in geriatric patients. Am J Med 1990; 88:101-7.
131. Lawrence VA, Dhanda R, Hilsenbeck SG, Page CP. Risk of pulmonary complications after elective abdominal surgery. Chest 1996; 110:744-50.
132. Norris EJ, Beattie C, Perler BA, et al. Double-masked randomized trial comparing alternate combinations of intraoperative anesthesia and postoperative analgesia in abdominal aortic surgery. Anesthesiology 2001; 95:1054-67.
133. Steinberg RB, Liu SS, Wu CL, et al. Comparison of ropivacaine-fentanyl patient-controlled epidural analgesia with morphine intravenous patient-controlled analgesia for perioperative analgesia and recovery after open colon surgery. J Clin Anesth 2002; 14:571-7.
134. Carli F, Mayo N, Klubien K, et al. Epidural analgesia enhances functional exercise capacity and health-related quality of life after colonic surgery: results of a randomized trial. Anesthesiology 2002; 97:540-9.
135. Jenkins K, Baker AB. Consent and anaesthetic risk. Anaesthesia 2003; 58:962-84.

136. Beattie WS, Choi PT. Canadian survey of the use of epidural anesthesia and analgesia. Can J Anesth 2003; 50:A109.
137. ICH Expert Working Group. ICH Harmonised Tripartite Guideline. Guideline for Good Clinical Practice. 1 May 1996.

Table 1. Meta-analyses of randomised clinical trials of perioperative drug therapy to reduce cardiac death and myocardial infarction in non-cardiac surgery.

Study	Studies	Cardiac Death			Myocardial Infarction		
		Treatment	Control	Effect (95% CI)*	Treatment	Control	Effect (95% CI)*
**b-adrenergic blockers**							
Stevens et al 2003 [69]	8	3 / 386	12 / 308	OR 0.25 (0.09-0.73)‡	3 / 349	17 / 324	OR 0.19 (0.08-0.48)‡
Devereaux et al 2004†	22	5 / 454	13 / 454	RR 0.40 (0.15-1.15)	21 / 442	37 / 412	RR 0.38 (0.11-1.28)
**Calcium channel blockers**							
Wijeysundera et al 2002 [84]	11	5 / 358	12 / 334	RR 0.40 (0.14-1.16)	0 / 252	5 / 234	RR 0.25 (0.05-1.18)
**Platelet inhibitors**							
Tangelder et al 1999 [90]	5	33 / 423	39 / 393	RR 0.71 (0.47-1.09)	25 / 351	34 / 312	RR 0.67 (0.41-1.09)
Robless et al 2001 [92]	10	46 / 893	53 / 872	OR 0.80 (0.53-1.21)	14 / 893	18 / 872	OR 0.71 (0.35-1.44)
Beattie et al 2002§	13	758 / 13987	790 / 13958	OR 0.95 (0.86-1.05)	162 / 8726	158 / 8718	OR 0.95 (0.76-1.16)
**a-agonists**							
Wijeysundera et al 2002 [100]	8¶	13 / 877	26 / 771	RR 0.47 (0.25-0.90)††	45 / 859	65 / 757	RR 0.66 (0.46-0.94)**
Wijeysundera et al 2002 [100]	3††	NR	NR	RR 1.05 (0.52-2.09)	NR	NR	RR 1.35 (0.83-2.21)
Stevens et al 2003 [69]	6	15 / 1357	29 / 1257	OR 0.51 (0.28-0.91)‡	81 / 1332	91 / 1242	OR 0.85 (0.62-1.14)
**Nitrates**							
Naik et al 2002‡‡	6	3 / 167	1 / 175	OR 0.42 (0.06-2.92)	0 / 352	4 / 264	OR 0.14 (0.02-1.24)

Abbreviations: CI, confidence interval; n/a, not applicable; NR, not reported; OR, odds ratio; RR, relative risk.

* An odds ratio or a relative risk <1 indicates a beneficial treatment effect; an odds ratio or a relative risk >1 indicates a harmful treatment effect. A 95% confidence interval that reaches or crosses over 1 indicates a non-significant effect.
† Devereaux PJ, Choi PT, Beattie WS, et al. How strong is the evidence for the use of perioperative beta-blockers in patients undergoing noncardiac surgery? A systematic review and meta-analysis. Manuscript submitted to Lancet. Includes data from the Metoprolol After Vascular Surgery (MAVS) Trial.
‡ $p<0.05$
§ Beattie WS, Naik J. Unpublished updated meta-analysis combining studies from Robless *et* al,[92] the ACE Trial,[86] Dutch Bypass Study,[87] and the PEP Trial.[92]
¶ Studies of patients undergoing vascular surgery.
** $p=0.02$
†† Studies of patients undergoing non-vascular non-cardiac surgery.
‡‡ Naik JS, Wijeysundera DN, Beattie WS, Choi PT. Unpublished manuscript.

Table 2. Meta-analyses of randomised clinical trials of perioperative interventions to reduce postoperative respiratory complications in non-cardiopulmonary surgery.

Study*	Studies	All Respiratory Complications			Postoperative Pneumonia		
		Treatment	Control	Effect (95% CI) †	Treatment	Control	Effect (95% CI) †
**Incentive spirometry**							
Thomas et al 1994 [103]	2	NR	NR	OR 0.44 (0.18-0.99)‡	NR	NR	NR
**Deep breathing exercises**							
Thomas et al 1994 [103]	4	NR	NR	OR 0.43 (0.27-0.63)§	NR	NR	NR
**Selective nasogastric decompression**							
Cheatham et al 1995 [104]	20	NR	NR	NR	51 / 1431	92 / 1502	RR 0.59 (NR)¶
**Selective digestive tract decontamination**							
Kollef 1994 [105]**	4	NR	NR	NR	16 / 231	80 / 242	OR 0.12 (0.06-0.21)§
D'Amico et al 1998 [106]**	9	NR	NR	NR	55 / 487	100 / 477	OR 0.51 (0.36-0.73)
Nathens et al 1999 [107]**	5	NR	NR	NR	11 / 233	41 / 243	OR 0.29 (0.14-0.58)§
**Nutritional support**							
Heys et al 1999 [108]**	4	NR	NR	NR	11 / 176	15 / 176	OR 0.71 (0.32-1.60)

Abbreviations: CI, confidence interval; n/a, not applicable; NR, not reported; OR, odds ratio; RR, relative risk.

* The control group was no physical therapy for incentive spirometry and deep breathing exercises; routine nasogastric decompress-ion for selective nasogastric decompression; placebo for selective digestive tract decontamination; and standard nutrition for nutritional support.

† An odds ratio or a relative risk <1 indicates a beneficial treatment effect; an odds ratio or a relative risk >1 indicates a harmful treatment effect. A 95% confidence interval that reaches or crosses over 1 indicates a non-significant effect.

‡ p=0.034

§ p=0.005

¶ p=0.01

** This meta-analysis evaluated mixed populations of critically ill patients. The values reported in this table are based only on surgical patients.

Table 3. Meta-analyses of randomised clinical trials and large randomised clinical trials of neuraxial blockade and perioperative cardiac events.

**Study**	**Studies**	**Death**			**Myocardial Infarction**		
		**NB**	**No NB**	**Effect (95% CI)***	**NB**	**No NB**	**Effect (95% CI)***
**Intraoperative blockade**							
Rodgers et al 2000[126]	141	103 / 4871	144 / 4688	OR 0.70 (0.54-0.90)†	45 / 4871	59 / 4688	OR 0.67 (0.45-1.00)
**Postoperative blockade**							
Beattie et al 2001[127]	11	18 / 579	26 / 585	OR 0.74 (0.40-1.37)	15 / 524	29 / 552	OR 0.58 (0.30-1.03)
Beattie et al 2003[128]	14				37 / 1156	59 / 1171	OR 0.64 (0.42-0.97)‡
Beattie et al 2003§	15				115 / 1603	134 / 1612	OR 0.84 (0.65-1.10)
**Perioperative blockade**							
Rodgers et al 2000[126]	NR	NR	NR	OR 0.68 (0.43-1.08)	NR	NR	NR
VA Cooperative Study[124]	1	20 / 514	17 / 507	OR 1.17 (0.60-2.25)	18 / 514	27 / 507	OR 0.65 (0.35-1.19)
MASTER Trial[125]	1	23 / 447	19 / 441	OR 1.20 (0.65-2.25)	78 / 447	75 / 441	OR 1.03 (0.73-1.46)**

Abbreviations: CI, confidence interval; NB, neuraxial blockade; NR, not reported; OR, odds ratio.

* An odds ratio <1 indicates a beneficial treatment effect; an odds ratio >1 indicates a harmful treatment effect. A 95% confidence interval that reaches or crosses over 1 indicates a non-significant effect.

† p=0.006

‡ p=0.03

§ Beattie WS, Choi PT, Badner NH. Updated meta-analysis with unpublished data on myocardial infarctions from the MASTER Trial (courtesy of Dr. John Rigg).

** Unpublished data on myocardial infarctions courtesy of Dr. John Rigg.

Table 4. Meta-analyses of randomised clinical trials and large randomised clinical trials of neuraxial blockade and perioperative respiratory events.

Study	Studies	Respiratory Failure			Pneumonia		
		**NB**	**No NB**	**Effect (95% CI)***	**NB**	**No NB**	**Effect (95% CI)***
**Intraoperative blockade**							
Rodgers et al 2000[126]	141	26 / 4871	38 / 4688	OR 0.41 (0.23-0.73)	149 / 4871	238 / 4688	OR 0.61 (0.48-0.76)
**Postoperative blockade**							
Ballantyne et al 1998[129]	5	NR	NR	NR	NR / 104	NR / 112	RR 0.36 (0.21-0.65)†
**Perioperative blockade**							
VA Cooperative Study[124]	1	51 / 514	71 / 507	OR 0.68 (0.46-1.00)	28 / 514	40 / 507	OR 0.67 (0.41-1.11)
MASTER Trial[125]	1	104 / 447	133 / 441	OR 0.70 (0.52-0.95)‡	NR	NR	NR

Abbreviations: CI, confidence interval; NB, neuraxial blockade; NR, not reported; OR, odds ratio; RR, relative risk.

* An odds ratio or relative risk <1 indicates a beneficial treatment effect; an odds ratio or relative risk >1 indicates a harmful treatment effect. A 95% confidence interval that reaches or crosses over 1 indicates a non-significant effect.

† p=0.0026

‡ p=0.02

Table 5. Rates of enrolment, follow-up, and crossover reported in randomised controlled trials of epidural *vs*. intravenous analgesia published in the past 10 years.

Study	Enrolment Period	Number of Patients	Number of Sites	Enrolment Rate (pt/site/month)	Complete Follow-up (%)	Crossover Rate (%)	
						Epidural to IV	IV to Epidural
Norris et al 2001[132]	Aug 1993 – Jul 1997	160	1	3.5	NR	NR	NR
VA Cooperative Study 2001[124]	NR	984	15	NR	973 / 984 (98.9)	32 / 489 (6.5)	48 / 495 (9.7)
Steinberg et al 2002[133]	Jul 1997 – Aug 1998	48	5	0.7	41 / 48 (85.4)	NR	NR
Carli F et al 2002[134]	Apr 1998 – Apr 2000	64	2	1.3	64 / 64 (100)	NR	NR
MASTER Trial 2002[125]	Jul 1995 – May 2001	920	25	0.5	NR	29 / 447 (6.5)	19 / 441 (4.3)

Abbreviations: IV, intravenous; NR, not reported.

Table 6. Use of intraoperative neuraxial blockade in hip and knee replacement surgeries in Canada during 2000 by geographical region, type of surgery, and type of hospital.*

	British Columbia	Alberta	Ontario	Other provinces†	Total
Academic hospitals	320 / 787 (39.9%)	1167 / 1780 (65.6%)	2227 / 5103 (43.6%)	1219 / 2753 (44.3%)	4933 / 10423 (47.3%)
Community hospitals	277 / 941 (29.4%)	160 / 834 (19.2%)	2314 / 7016 (33.0%)	590 / 1867 (31.6%)	3341 / 10658 (31.3%)

* Choi PT, Devereaux PJ, Weaver B, Guyatt GH. Unpublished analysis of data from the Canadian Institute of Health Information.

† Excludes Quebec and rural Manitoba.

Table 7. Epidural and IV analgesic formulations currently used by the Acute Pain Services of the five participating sites of the POET Pilot Study.

**Epidural local anesthetic / narcotic mixtures**	
Hamilton Health Sciences	Bupivacaine 0.125% + fentanyl 5 mcg/mL<br>Bupivacaine 0.125% + morphine 50 mcg/mL
Ottawa Hospital	Bupivacaine 0.1% + fentanyl 2 mcg/mL<br>Bupivacaine 0.1% + hydromorphone 10 mcg/mL<br>Bupivacaine 0.1% + hydromorphone 20 mcg/mL
University Health Network*	Bupivacaine 0.1% + fentanyl 4 mcg/mL<br>Bupivacaine 0.1% + hydromorphone 15 mcg/mL
Vancouver Hospital	Bupivacaine 0.1% + morphine 50 mcg/mL<br>Bupivacaine 0.2% + morphine 25 mcg/mL<br>Bupivacaine 0.1% + hydromorphone 20 mcg/mL<br>Bupivacaine 0.2% + hydromorphone 10 mcg/mL
**Intravenous narcotics**	
Hamilton Health Sciences	Morphine 5 mg/mL
Ottawa Hospital	Morphine 2 mg/mL<br>Meperidine 20 mg/mL<br>Hydromorphone 0.4 mg/mL
University Health Network*	Morphine 2 mg/mL
Vancouver Hospital	Morphine 5 mg/mL<br>Meperidine 10 mg/mL<br>Hydromorphone 1 mg/mL

* The Toronto General Hospital and Toronto Western Hospital are both part of the University Health Network and use the same drug formulations.

Table 8. Eligibility criteria for the PeriOperative Epidural Trial Pilot Study.

Inclusion Criteria

Any patient undergoing non-cardiopulmonary surgery and

1. is =45 years old;
2. has an expected length of stay =48 hours;
3. is undergoing a procedure amenable to postoperative epidural analgesia *(i.e. major lower limb, vascular, retroperitoneal, intraperitoneal, or intrathoracic procedures)*; AND
4. fulfills any of the following six criteria
   i. history of coronary artery disease *(defined as history of angina, prior MI, prior positive exercise stress test, prior documentation of cardiac ischemia on nuclear stress testing, prior coronary angiographic evidence of atherosclerotic stenosis >50% of vessel diameter, or ECG with pathological Q-waves in two contiguous leads)*;
   ii. history of peripheral vascular disease *(defined by any of the following: leg pain on walking that disappears in <10 minutes and is known or likely to be due to atherosclerotic disease, an ankle/brachial systolic blood pressure ratio =0.90 in either leg at rest, or angiographic or Doppler evidence of >70% stenosis)*;
   iii. history of atherothrombotic stroke *(defined as a focal neurological deficit persisting for =1 week after onset that is not a lacunar stroke, hemorrhagic stroke, or embolic stroke)*;
   iv. hospitalization for congestive heart failure (CHF) within three years of randomisation;
   v. undergoing major vascular surgery *(i.e. any vascular procedure excluding arteriovenous dialysis shunts and varicose vein procedures)*; OR
   vi. has at least three of the following factors
      a. any history of CHF,
      b. diabetes currently requiring oral hypoglycemic or insulin therapy,
      c. history of transient ischemic attack *(i.e. a transient focal neurologic deficit of vascular origin lasting <24 hours)*,
      d. history of chronic obstructive pulmonary disease *(defined as chronic obstruction to airflow based on best spirometry within past 12 months [FEV_1 <60% and FEV_1/FVC ratio <75% of predicted values] that is not due to asthma AND results in dyspnea on walking =2 blocks, previous hospitalisation to treat the disease, or need for regular bronchodilator or steroid therapy with oral or inhaled agents)*;
      e. preoperative serum creatinine >175 µmol/L,
      f. age >70 years,
      g. anticipated duration of anesthesia =3 hours,
      h. intraperitoneal or intrathoracic surgery,
      i. surgery that must be undertaken within 24 hours of acute presentation to hospital

---

Table 8. Eligibility criteria for the PeriOperative Epidural Trial Pilot Study (continued).

Exclusion Criteria

1. contraindication to epidural analgesia
   i. stable platelet count <50,000 mm^{-3} or a falling platelet count <100,000 mm^{-3};
   ii. abnormal INR or aPTT;
   iii. ongoing use or planned peri-operative use of anticoagulants *(e.g. ticlopidine, coumadin, heparin, dalteparin)*;
   iv. systemic infection with elevated white blood cell count and temperature >37.5 ^{o}C;
   v. local infection at proposed site for epidural insertion;
   vi. severe cardiac valvular abnormalities that do not tolerate afterload reduction *(e.g. severe aortic stenosis or severe mitral stenosis)*;
   vii. vertebral abnormalities that prevent proper placement of an epidural catheter or spread of epidural drugs *(e.g. spinal instrumentation)*;
2. prior adverse reaction to local anesthetics or narcotics;
3. previous coronary artery bypass graft surgery with complete revascularisation in the preceding five years AND no evidence of cardiac ischemia since the procedure;
4. pneumonia *(defined as an infiltrate on chest radiograph and / or positive sputum cultures with initiation of antibiotic therapy)* within two weeks of surgery;
5. currently intubated or mechanically ventilated;
6. concomitant life-threatening disease likely to limit life expectancy to <30 days *(e.g. palliative resection of obstructive tumours)*

Table 9. Definition of clinical study outcomes to be used if a large multicentre trial is conducted.

**Primary Outcome**	
Death	Any death regardless of cause
Nonfatal myocardial infarction	A typical rise of troponin OR a typical fall of an elevated troponin OR a rapid rise and fall of CK-MB and one of the following:<br>1) characteristic ischemic symptoms;<br>2) development of pathological Q waves on ECG;<br>3) ECG changes indicative of ischemia;<br>4) coronary artery intervention; OR<br>5) new or presumed new cardiac wall motion abnormality on echocardiographic or radionuclide imaging<br><br>OR<br><br>Pathological findings of acute myocardial infarction
Cardiac arrest	Any successful resuscitation from a documented or presumed ventricular fibrillation OR sustained ventricular tachycardia OR asystole
Clinically significant postoperative pneumonia	Any condition with:<br>1) fever (temperature > 38.0 degrees Celsius), leukopenia (<4000 wbc/mm^3), leukocytosis (=12000 wbc/mm^3), OR (in adults =70 years old) altered mental status; AND<br>2) two of the following: a) new onset of purulent sputum or change in sputum character, b) new onset of worsening cough, dyspnea, or tachypnea, c) rales or bronchial breath sounds organism isolated from blood culture, or d) O_2 desaturation [PaO_2/FiO_2 =240], increased O_2 requirements, or increased ventilation demand; AND<br>3) two or more serial chest radiographs with new or progressive persistent infiltrate, consolidation, or cavitation
Respiratory failure	Any condition requiring intubation of the trachea and mechanical ventilation AFTER completion of surgery, emergence from anesthesia, successful extubation (if intubated during surgery), and spontaneous ventilation for =1 h after surgery

Table 9. Definition of study outcomes (continued).

**Secondary Outcomes**	
Deep vein thrombosis	Any clinical suspicion of DVT (lower limb pain OR tenderness OR swelling OR edema) AND objective diagnostic confirmation (positive lower limb venogram with constant intraluminal filling defect seen on =2 views OR compression ultrasound demonstrating a noncompressible vein segment)
Pulmonary embolus	Any clinical suspicion of PE (chest pain OR shortness of breath) AND objective diagnostic confirmation (definite PE = a pulmonary angiogram with a constant intraluminal filling defect OR a spiral computed tomogram with an unenhanced filling defect seen in a central pulmonary artery OR a high-probability VQ scan OR an intermediate VQ scan with venographic evidence of DVT OR autopsy evidence of PE; probable PE = an intermediate VQ scan with clinical signs)
Transient ischemic attack	Any new focal neurological deficit of vascular origin that lasts <24 h with no permanent neurological sequelae
Stroke	Any new focal neurological deficit of vascular origin with signs and symptoms lasting =24 h
Congestive heart failure	Any condition with both clinical (elevated jugular venous pressure OR respiratory rales OR crepitations OR presence of S3) AND radiological (vascular redistribution OR interstitial pulmonary edema OR frank pulmonary edema) evidence consistent with CHF
**Safety Outcomes**	
Clinically important bradycardia	Heart rate <60 bpm requiring temporary pacemaker, sympathomimetic agent, or atropine
Clinically important hypotension	Systolic blood pressure that is at least 20% lower than the preoperative SBP AND requires fluid resuscitation, a vasopressor, or an inotropic agent

Abbreviations: bpm, beats per minute; DVT, deep vein thrombosis; ECG, electrocardiogram; LBBB, left bundle branch block; PE, pulmonary edema; SBP, systolic blood pressure; VQ, ventilation-perfusion

Table 10. Sample size calculations for a large multicentre randomised controlled trial.

Relative Risk Reduction	Control Event Rate (Type I Error = 5%; Power = 90%)		
	**15%**	**20%**	**25%**
**20%**	5452	3874	2928
**25%**	3406	2424	1836
**30%**	2306	1644	1246

Relative Risk Reduction	Control Event Rate (Type I Error = 5%; Power = 80%)		
	**15%**	**20%**	**25%**
**20%**	4072	2894	2188
**25%**	2544	1812	1372
**30%**	1722	1228	932

All calculations are based on a combined 30-day outcome of all-cause mortality, nonfatal myocardial infarction, cardiac arrest, postoperative pneumonia, and respiratory failure. The sample size refers to the total number of patients required for the entire study but does not account for losses to follow-up or crossovers between groups.

Figure 1. Frequency of Combined Cardiac Death and Myocardial Infarction in Prospective Studies of Non-Cardiac Surgery

STUDY (CARDIAC EVENTS)		NUMBER	% (95% CI)
Goldman	1977	1001	
Zeldin	1984	1140	
Larsen	1987	2609	
Pedersen	1994	7306	
Lee	1999	4315	
CONSECUTIVE		16371	1.8 (1.8; 1.8)
Detsky	1986	455	
Mangano	1990	474	
Shah	1990	688	
Ashton	1993	835	
Gilbert	2000	2035	
CONSECUTIVE + CRITERIA		4487	3.7 (3.6; 3.7)
Lette	1992	355	
Brown	1993	231	
Halm	1996	339	
CONSECUTIVE + TESTING		925	5.4 (5.3; 5.6)
ALL CONSECUTIVE		21783	3.3 (3.3; 3.3)
McPhail	1985	353	
Raby	1989	176	
Pasternack	1992	385	
Raby	1992	115	
Jivegard	1993	235	
Landesberg	1993	151	
VASCULAR		1415	5.9 (5.9; 6.0)
Hendel	1992	327	
Davila-Roman	1993	98	
McFalls	1993	116	
Poldermans	1993	136	
Baron	1994	457	
Bry	1994	237	
L'Italien	1996	1081	
VASCULAR + TESTING		2452	7.5 (7.5; 7.6)
ALL VASCULAR		3867	6.8 (6.7; 6.8)
ALL PATIENTS		25650	5.0 (5.0; 5.0)

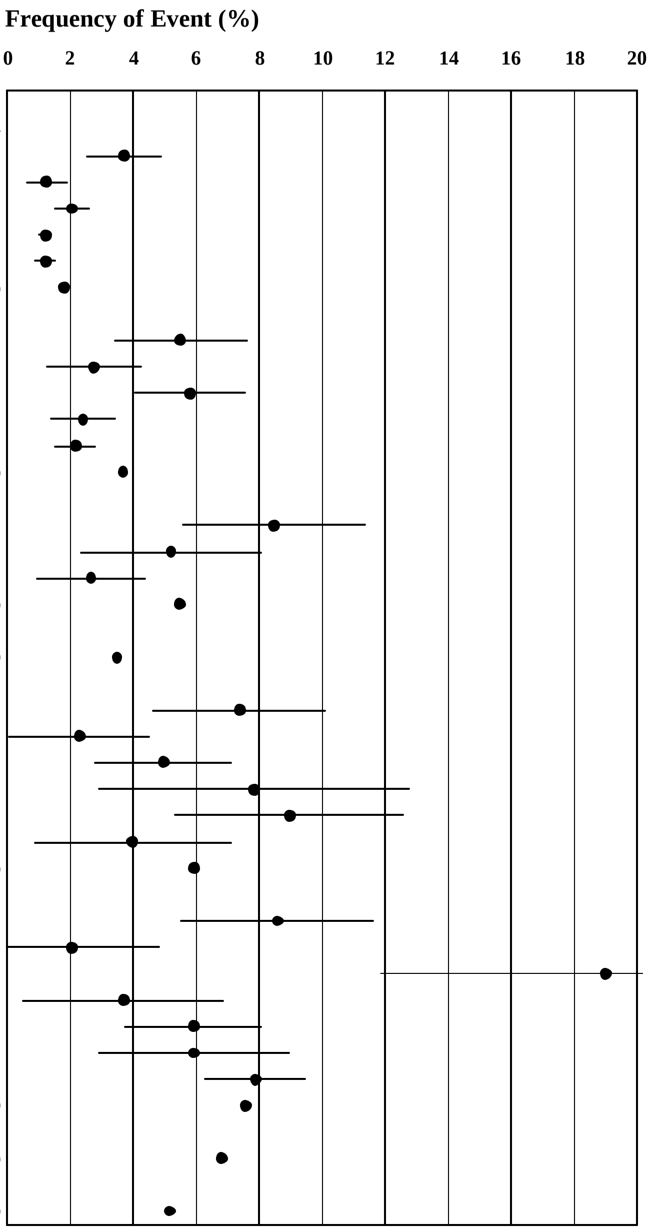

Figure 2. Frequency of Postoperative Pneumonia in Prospective Studies of Non-cardiopulmonary Surgery

STUDY (PNEUMONIA)		NUMBER	% (95% CI)
Wightman	1968	785	
Ti	1974	663	
Appleberg	1974	100	
Jarhult	1981	1409	
Pedersen	1990	7306	
CONSECUTIVE		10263	2.5 (2.5; 2.5)
Wong	1995	105	
Mitchell	1998	148	
Arozullah	2001	160805	
de Albuquerque	2001	59	
CONSECUTIVE + CRITERIA		161117	8.0 (8.0; 8.1)
ALL CONSECUTIVE		171380	5.0 (5.0; 5.0)
Daschner	1982	2044	
Donowitz	1982	1246	
Kappstein	1991	418	
Konrad	1991	582	
CRITICALLY ILL		4290	12.5 (12.4; 12.6)
Latimer	1971	46	
Grundmann	1987	3539	
Lumbreras	1992	50	
Williams-Russo	1992	278	
Ephgrave	1993	140	
Lawrence	1995	2291	
Liedman	1995	132	
Hall	1996	1332	
Brooks-Brunn	1997	400	
Delgado-Rodriguez	1997	1483	
Mathew	1999	484	
Whooley	2001	710	
ABDOMINAL		10885	13.7 (13.7; 13.8)
Johnston	1989	666	
Svensson	1991	100	
VASCULAR		766	4.8 (4.6; 5.0)
ALL HIGH-RISK		15941	12.5 (12.4; 12.5)
ALL PATIENTS		187321	10.0 (10.0; 10.0)

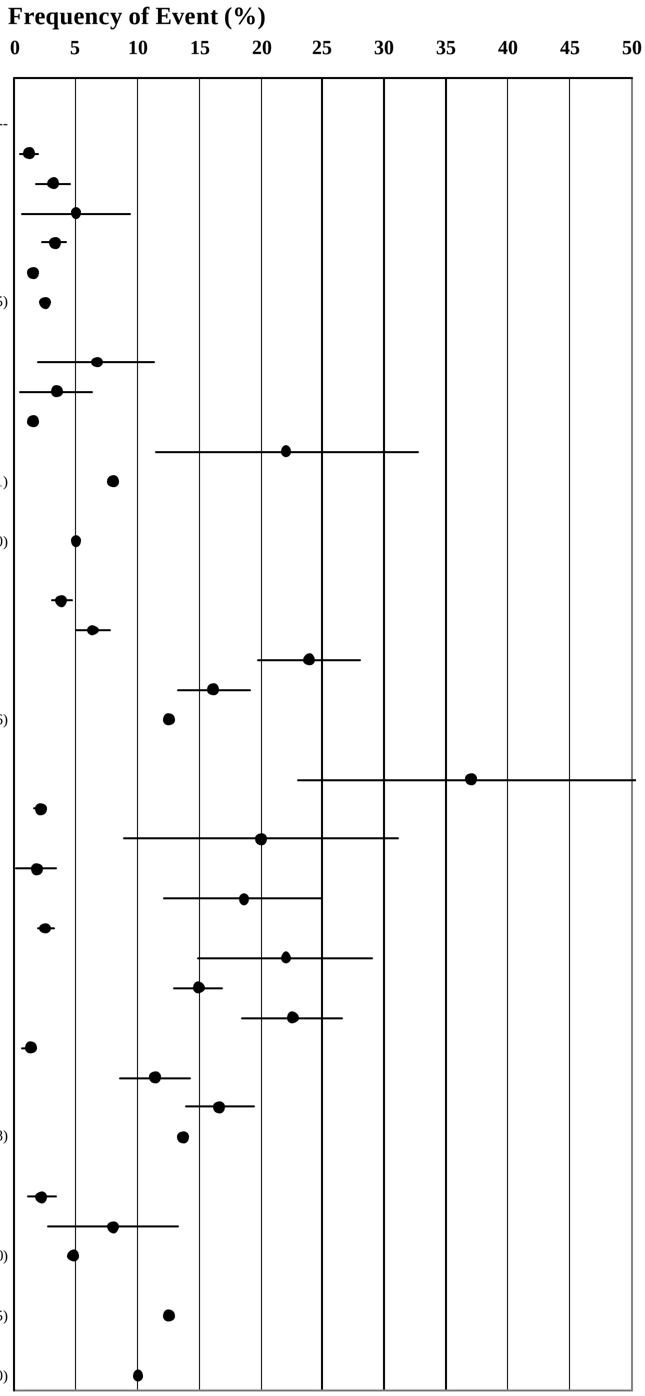

Figure 3. Diagram of neuraxial anatomy. (Figure taken from Brown 2000.[111])

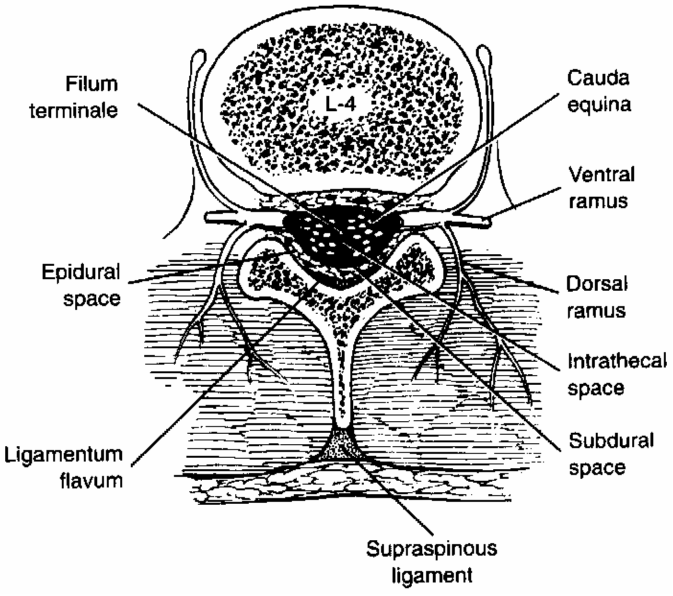

Figure 4. Study flow chart.

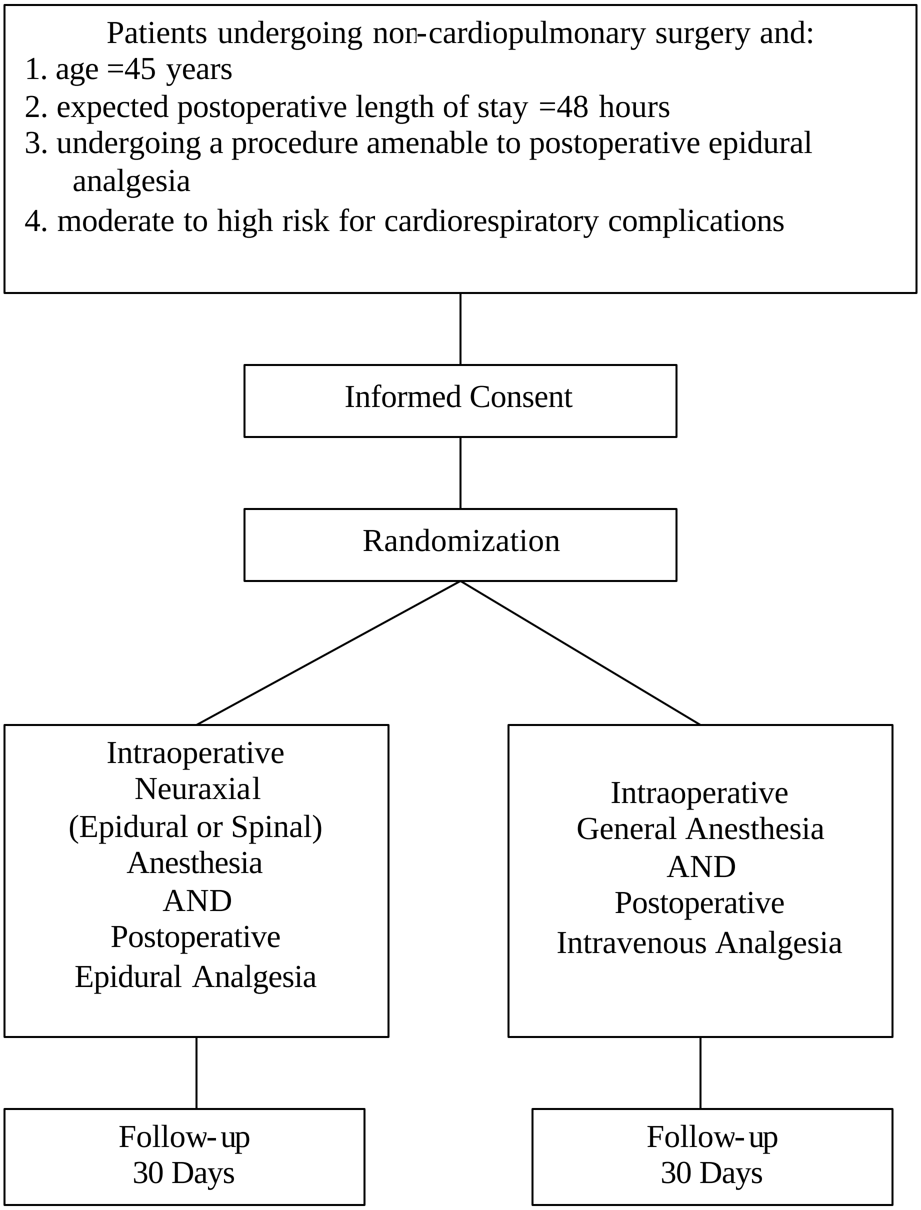

Figure 5a. Photograph showing a setup of an epidural infusion pump and an intravenous patient-controlled narcotic analgesia pump for the POET Pilot Study.

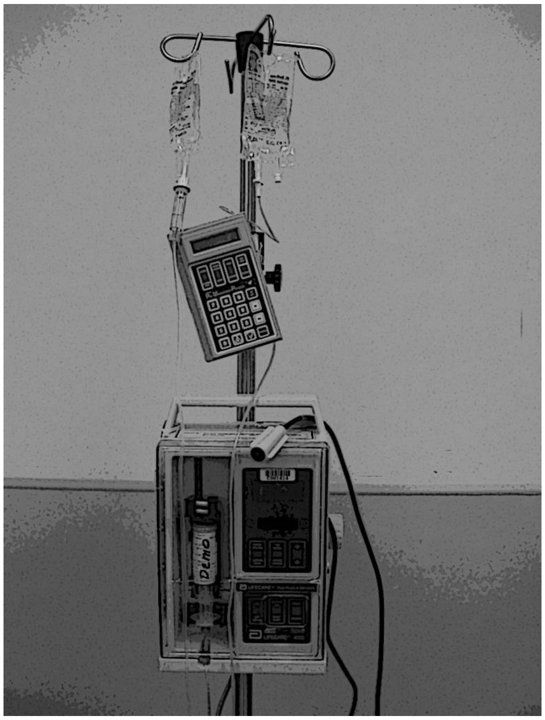

Figure 5b. Photograph showing the masking of the analgesic pumps and the epidural and IV tubings when the blinded investigator is conducting a clinical assessment of the patient.

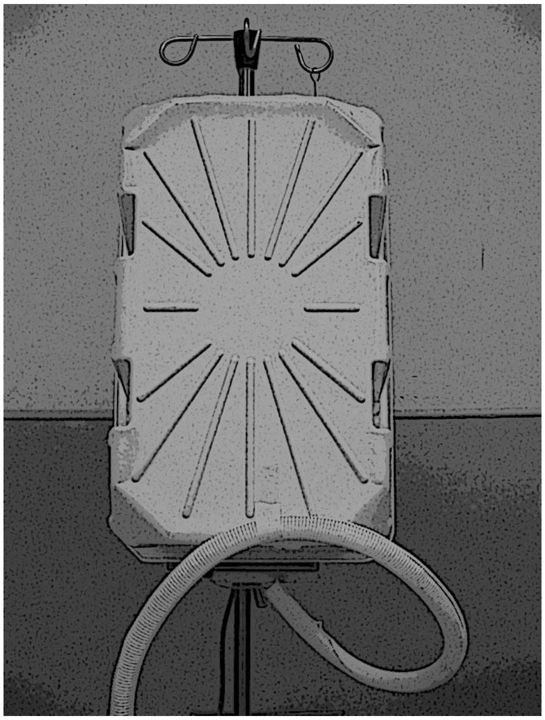

Supplement: Protocol S1 — Trial Protocol (1.23 MB PDF) [file pone.0004644.s001.pdf]
