# Supplementary material for: Effects of Neuraxial Blockade May Be Difficult To Study Using Large Randomized Controlled Trials: The PeriOperative Epidural Trial (POET) Pilot Study
Source: PLoS One. 2009 Feb 27;4(2):e4644. doi: 10.1371/journal.pone.0004644 (PMC2645707; doi:10.1371/journal.pone.0004644)
Supplement: Contributions S1 — Contributions of participants in the POET Pilot Study (0.04 MB DOC) [file pone.0004644.s003.doc]

The following individuals participated in the POET Pilot Study:

**Steering Committee:** W. Scott Beattie (co-principal investigator)**^2^**, D. Norman Buckley**^4^**, Vincent W.S. Chan**^2^**, Peter T. Choi (project leader & co-principal investigator)**^1^**, Ashraf Fayad**^3^**, York Hsiang**^5^**, Homer Yang**^3^**

**Central Coordinating Office:** Penelope Brasher (statistician)**^6^**, Keith Chambers**^6^**, Peter T. Choi**^1^**, Cheryl Davies (coordinator)**^6^**, Bobby Sidhu (programmer analyst)**^6^**

**Sites:** *Hamilton Health Sciences* – James E. Paul (site investigator)**^4^**, Lynda Rickards**^4^**, *Ottawa Hospital* – Gregory L. Bryson (site investigator)**^3^**, Anne Lui**^3^**, John Penning**^3^**, Sharon Finlay**^3^**, Denise Winch**^3^**, *University Health Network* – W. Scott Beattie**^2^**, Duminda N. Wijeysundera**^2^**, Jo Carroll**^2^**, Humera Poonawala**^2^**, Filomena Valle**^2^**, *Vancouver General Hospital* – Hamed Umedaly (site investigator)**^7^**, Marion Eng**^7^**, Matthew Iype**^7^**

**Events Adjudication Committee:** Vincent W.S. Chan**^2^**, Philip J. Devereaux**^8^**, J. Mark Fitzgerald**^9^**, York Hsiang**^5^**

**Data Safety Monitoring Committee:** J. Mark Ansermino**^1^**, Najib Ayas**^9^**, Ruth Milner**^10^**, Graham Wong**^11^**

**1** Department of Anesthesiology, Pharmacology and Therapeutics, University of British Columbia, Vancouver, British Columbia, Canada, **2** Department of Anaesthesia, University Health Network, Toronto, Ontario, Canada, **3** Department of Anesthesiology, University of Ottawa, Ottawa, Ontario, Canada, **4** Department of Anesthesia, McMaster University, Hamilton, Ontario, Canada, **5** Division of Vascular Surgery, University of British Columbia, Vancouver, British Columbia, Canada, **6** Centre for Clinical Epidemiology and Biostatistics, Vancouver Coastal Health Research Institute, Vancouver, British Columbia, Canada, **7** Department of Anesthesia, Vancouver General Hospital, Vancouver, British Columbia, Canada, **8** Department of Clinical Epidemiology and Biostatistics, McMaster University, Hamilton, Ontario, Canada, **9** Division of Respiratory Medicine, University of British Columbia, Vancouver, British Columbia, Canada, **10** Department of Biostatistics, BC Children’s Hospital, Vancouver, British Columbia, Canada, **11** Division of Cardiology, University of British Columbia, Vancouver, British Columbia, Canada
